# Reversible Phase Transitions of Anionic and Cationic Surfactant Mixtures Drive Shape Morphing Droplets

**DOI:** 10.1002/adma.202506100

**Published:** 2025-06-26

**Authors:** Bradley D. Frank, Pilar Romero, Alberto Concellón, Lukas Zeininger

**Affiliations:** ^1^ Department of Colloid Chemistry Max Planck Institute of Colloids and Interfaces Am Mühlenberg 1 14476 Potsdam Germany; ^2^ Instituto de Nanociencia y Materiales de Aragón (INMA) CSIC‐Universidad de Zaragoza Zaragoza ES‐50009 Spain

**Keywords:** active matter, droplets, liquid crystals, out‐of‐equilibrium, stimuli‐responsive surfactants, soft robotics

## Abstract

Converting chemical signals into mechanical responses is fundamental to biological systems, driving processes such as cellular motility and tissue morphogenesis. Yet, harnessing chemo‐mechanical signal conversions in synthetic systems remains a key challenge in energy‐dissipative materials design. While droplets can move and interact with their environment reminiscent of active biological matter, chemo‐mechanical interactions are limited by the translation of chemical changes into extensive force variations required on small timescales. Droplets naturally adopt spherical shapes to minimize surface‐energy and restructuring liquids into non‐equilibrium geometries requires mechanisms beyond current stimuli‐responsive surfactant systems, which lack the force‐amplifying mechanisms needed for transient liquid structuring. Here, a spring‐like charging and latch‐controlled release mechanism is introduced for actuating droplets. This is based on reversible, light‐induced crystal‐to‐coacervate phase transitions of photo‐responsive surfactant assemblies, namely between anionic sodium dodecylsulfate and cationic azobenzene‐based surfactants. During phase‐transition, reversible partitioning of the surfactants into the oil or aqueous phases of the emulsion transiently induce rapid changes in interfacial tensions, which are up to 900 times greater than those observed for conventional stimuli‐responsive surfactant systems. The insights into this novel chemo‐mechanical transduction mechanism provide new control over purely liquid systems, paving the way for programmable, hierarchically structured, all‐liquid matter acting with physicality.

## Graphical abstract

A light‐controlled, reversible phase transition of ionic surfactant assemblies, coupled with dynamic partitioning between bulk phases and interfaces, induces rapid, and order‐of‐magnitude changes in oil–water interfacial tension. Surpassing the capabilities of conventional stimuli‐responsive surfactants, this transient, collaborative mechanism enables multimodal droplet shape transformations and offers a new strategy for the design of programmable, hierarchically structured, all‐liquid matter acting with physicality.

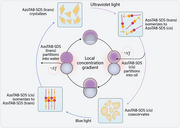

## 1 Introduction

Biological systems ubiquitously interact with and move in response to their environment, often employing chemo‐mechanical mechanisms to swim, jump, or push.^[^ ^1^, ^2^, ^3^ ^]^ While life utilizes varied energetic pathways to convert chemical energy into mechanical responses, inanimate liquid matter systems have yet to fully address the conversion of chemical energy into tactile modes of interaction.^[^ ^4^, ^5^ ^]^ Emulsion droplets offer a robust platform for both passive and active matter, emulating energy‐dissipative natural systems by controlling energy transfer through active chemistries and discharging energy gradients.^[^ ^6^, ^7^, ^8^ ^]^ This enables them to move, adapt, and interact with their chemical environment in a manner reminiscent of active biological matter. However, multi‐scale and reversible chemo‐mechanical interactions remain limited by the challenge of translating chemical changes into extensive force variations on small timescales.

The distribution of oil, water, and surfactants inside a macroemulsion is highly dynamic, with droplets continuously changing composition and volume over time. As a result, they possess an acute sense of their immediate chemical environment. Exploiting this dynamic, out‐of‐equilibrium behavior has allowed the generation of synthetically minimalistic droplet systems that exhibit simple, yet sophisticated emergent collective behaviors such as a self‐regulated ability to sense, move, communicate, evolve, and organize into patterns or networks.^[^ ^9^, ^10^, ^11^, ^12^ ^]^ Through energy dissipation, synthetic liquid systems have been shown to self‐propel,^[^ ^13^ ^]^ become sensitive to chemical gradients,^[^ ^14^ ^]^ sometimes generated by nearby droplets,^[^ ^15^, ^16^, ^17^ ^]^ respond to thermal or chemical gradients,^[^ ^18^, ^19^ ^]^ and can change shape and self‐propel by undergoing rechargeable morphological transitions.^[^ ^20^ ^]^ By targeting different responsive modalities of droplets, adaptivity can be achieved, enabling droplets to effectively transduce molecular information from their environment into macroscopically detectable signals, often facilitated by stimuli‐responsive surfactants.^[^ ^21^, ^22^, ^23^, ^24^ ^]^ Yet, multi‐scale and reversible chemo‐mechanical interactions are limited by the translation of chemical changes into the extensive force variations required on small timescales.

Droplets naturally adopt spherical shapes to minimize surface energy and restructuring liquids into non‐equilibrium geometries requires new mechanisms for rapid and significant power amplification beyond current stimuli‐responsive surfactant systems, which are constrained by modest interfacial tension variations and lack the force‐amplifying mechanisms needed for transient liquid structuring. Surface‐active agents play a central role in emulsion manipulation, primarily by reducing the interfacial tension between the two liquid phases. Classical stimuli‐responsive emulsions primarily work through externally evoked variations in surfactant hydrophobicity, thereby increasing or lowering interfacial tensions and affecting droplet stability. Stimuli‐responsive surfactants, such as light‐responsive azobenzene‐based surfactants are broadly employed for dynamically actuating droplets in response to external physical or chemical triggers.^[^ ^25^, ^26^, ^27^ ^]^ They are, however, constrained by modest interfacial tension variations (typically on the order of 5–10 mN m^−1^), which are discharged continuously, and lack a force‐amplifying mechanism needed for transiently structuring liquids and overcoming the force equilibrium of low‐energy spherical droplets.

As opposed to continuously dissipating energy or gradients, many power‐amplified systems in nature, including both animals and plants store energy in spring‐latch elements allowing them to release the energy on demand.^[^ ^28^, ^29^, ^30^, ^31^ ^]^ Consequently, we hypothesized that an efficient power‐amplified chemo‐mechanical actuation of droplets requires mechanisms integrating a motor, spring, and latch component to enhance energy output to enable tactile droplet actuations. To maximize power densities of elastic liquid materials, such charge/discharge mechanisms necessitate new mechanisms to activate emulsion droplets, e.g., via kinetic trapping of non‐equilibrium droplet geometries and utilization of rapid chemical energy dissipation associated with order‐of‐magnitude variations in interfacial tension to enable a rapid second‐scale droplet shape actuation.

Here, we introduce a novel stimuli‐responsive surfactant system capable of achieving order‐of‐magnitude changes in interfacial tension within seconds, thereby enabling the transient structuring and shape morphing of purely liquid matter. In our approach, we leverage a reversible precipitation of surfactant assemblies from the continuous phase, introducing a spring‐like charging and latch‐controlled release mechanism for actuating droplets. The mechanism is based on a reversible, light‐induced crystal‐to‐coacervate phase transition of a photo‐responsive surfactant assembly between anionic sodium dodecyl sulfate and cationic azobenzene‐based surfactants. During phase transition, the reversible partitioning of surfactant assemblies into the oil or aqueous phases of the emulsion transiently induces rapid, order‐of‐magnitude changes in interfacial tension. This enables unprecedented multimodal shape reconfigurations of droplets on demand, including linear actuation, destruction, and reformation within seconds. Insights into this novel chemo‐mechanical transduction mechanism provide new control over purely liquid systems, paving the way for programmable, hierarchically structured, all‐liquid matter capable of acting with physicality.

## 2 Results and Discussion

**Figure 1:**
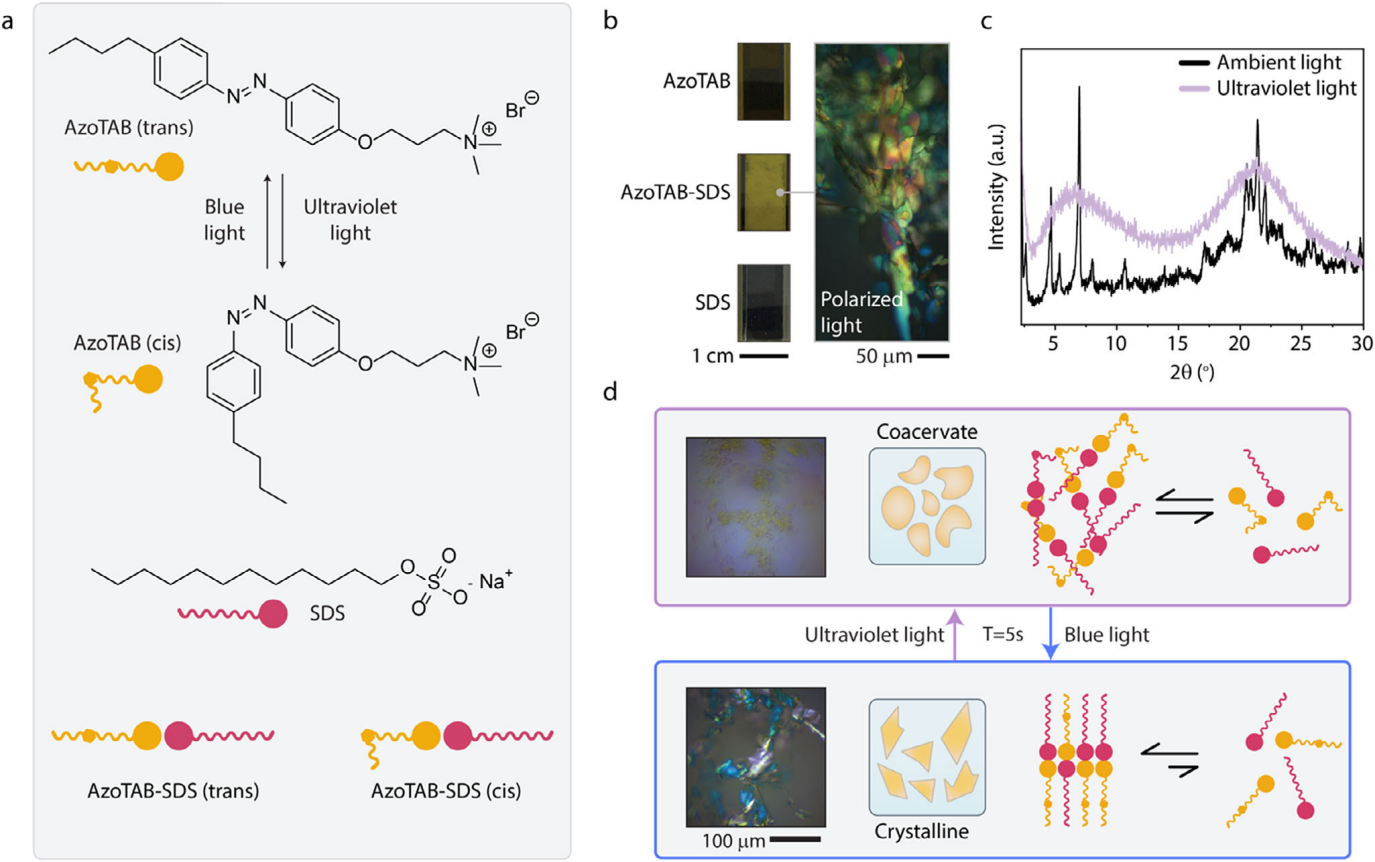
AzoTAB and SDS form a supramolecular assembly with reversible crystallinity. a) Chemical structure of the cationic surfactant AzoTAB with scheme of *cis‐trans* photoisomerization, alongside the chemical structure of the anionic hydrocarbon‐surfactant sodium dodecyl sulfate (SDS). b) Images of vials containing pure SDS, pure AzoTAB, and a 1:1 mixture of AzoTAB and SDS (5 mM), with a polarized optical micrograph of the crystalline precipitate formed by AzoTAB‐SDS. c) XRD of AzoTAB‐SDS under ambient lighting (polycrystalline) and under ultraviolet light (melted state). d) Scheme for the crystalline‐to‐coacervate transition between SDS‐AzoTAB (*cis*) and SDS‐AzoTAB (*trans*).

Mixing solutions of two surfactants, the anionic sodium dodecyl sulfate (SDS) and the cationic light‐responsive azobenzene trimethylammonium bromide (AzoTAB) (**Figure** **1**a) at equimolar concentrations under ambient light conditions causes the aqueous mixture to become turbid within minutes, reflecting surfactant precipitation. Under polarized light microscopy the precipitate appears as a birefringent crystal (Figure 1b; Figures S1 and S2, Supporting Information). X‐Ray diffraction (XRD) patterns of the birefringent crystal precipitate display several sharp reflections, indicating a polycrystalline sample. The rod‐shape structure of *trans*‐AzoTAB promotes robust lateral π–π interactions between AzoTAB‐SDS assemblies, fixing their position and resulting in lack of mobility essential for the emergence of crystal ordering. AzoTAB is a light‐responsive surfactant consisting of an azobenzene moiety that can undergo a reversible photo‐induced *cis‐trans* isomerization depending on the incident wavelength.^[^ ^32^, ^33^ ^]^ Upon exposure of the AzoTAB‐SDS precipitate to ultraviolet light, the XRD pattern exhibited only diffuse scattering, suggesting the absence of long‐range positional order in this second phase (Figure 1c). The observed behavior can be attributed to the bent shape of the *cis*‐AzoTAB hindering strong lateral interactions that promote the crystallization of *trans*‐AzoTAB, yielding an amorphous analog and resulting in melting into an isotropic liquid state. This phase behavior is also observed through consecutive heating and cooling cycles, indicating that the supramolecular assembly of AzoTAB‐SDS corresponds to a thermotropic nematic liquid crystal (Figure S3, Supporting Information). The melted precipitate under UV‐light exposure yields a fluid water‐insoluble secondary phase that can be attributed to as a stoichiometric zero‐charge coacervate and subsequent application of blue light resulted in rapid crystallization back to the initial crystalline state within ≈5 s (Figure 1d and Video S1, Supporting Information). These results were replicable with heating and cooling (Figures S2 and S3, Supporting Information). The change of the birefringence between crystalline and coacervated states indicates the topologically disordered and ordered states of the supramolecular assembly, where *cis‐*AzoTAB in AzoTAB‐SDS hinders an ordered structure, while an intermediary nematic liquid crystal is visible in between the pure crystalline and the pure melted states (Figure S3 and Video S2, Supporting Information).

NMR spectroscopy experiments support our finding that supramolecular assemblies of anionic and cationic surfactants precipitate depending on their ratio and concentration.^[^ ^34^, ^35^ ^]^ Below the CMC of either surfactant, precipitate and monomeric surfactant molecules exist in equilibrium, with additional equilibria between monomeric and micellar states above the CMC. ^1^H‐^1^H nuclear overhauser effect spectroscopy (NOESY) NMR experiments showed that micelles of AzoTAB integrate monomeric SDS molecules, which interact with water molecules (Figures S4 and S5, Supporting Information). In our experiments, by using a photo‐switchable azo‐benzene‐based surfactant in an equimolar mixture with the anionic surfactant, the *cis‐trans* isomerization is not only modifying the effectiveness and solubility of AzoTAB as an oil‐water surfactant, but additionally alters the precipitate morphology between an amorphous liquid or solid crystalline state in water. In switching AzoTAB‐SDS between the *cis* and *trans* conformation, the equilibrium between precipitate, and monomer is changed, as verified through titration‐NMR, and supported by the similar diffusion coefficient of monomeric AzoTAB, SDS, and *cis*‐AzoTAB‐SDS in diffusion ordered NMR spectroscopy (DOSY‐NMR) (Figures S6 and S7 and Table S1, Supporting Information).

At room temperature and ambient light conditions AzoTAB predominantly exists in its *trans* conformer (ratio *trans:cis*: ≈95:5 % as determined by NMR (Figure S8, Supporting Information), and hence yields a solid precipitate when mixed with SDS. Upon irradiatation with UV light, isomerization in pure AzoTAB solutions occurs in less than 1 min (Figure S9, Supporting Information). This indicates that marginal shifts in the *trans:cis* isomer ratio suffice to induce the phase transitions of the AzoTAB‐SDS assembly under our lightning conditions.

Having established the reversible crystal‐to‐coacervate transition of the surfactant assembly in pure water, direct interaction of AzoTAB‐SDS with biphasic liquid systems was used to investigate the effects on interfacial stabilization that changing surfactant equilibria have on the system. Given the lipophilic nature of the liquid surfactant assembly under UV light, we investigated its partitioning behavior in a biphasic oil–water system by adding the surfactants to the mixture.

**Figure 2:**
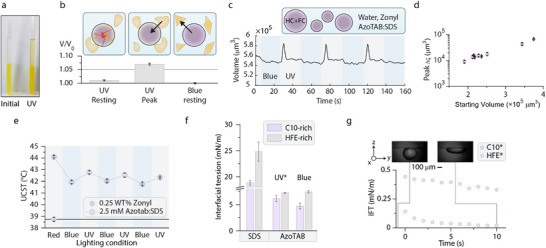
AzoTAB‐SDS assemblies show switchable partitioning behavior in oil and water mixtures, which creates transient local interfacial tension gradients. a) Photograph of visible change in phase partitioning of the SDS‐AzoTAB assembly in an oil‐water system under different lighting conditions. b) Comparable change in volume for a single droplet over multiple actuations, displaying the three states of volume change, due to blue light, ultraviolet light, and the observable peak. c) Volume versus time for a single C8:HFE droplet measured under application of blue and ultraviolet light in equimolar SDS‐AzoTAB. d) Change in volume versus starting volume measured for room‐temperature miscible droplets of octane and HFE‐7500 in equimolar solutions of AzoTAB‐SDS, measured from the resting and peak volume of polydisperse droplets. e) Upper critical solution temperature (UCST) of C10:HFE droplets in equimolar AzoTAB‐SDS under ultraviolet and blue illumination versus droplets in pure Zonyl solutions. f) Pendant drop tensiometry of either C10‐rich, or HFE‐rich oils versus noted aqueous solutions. g) Interfacial tension over time for liquid sessile droplets of either decane‐rich or HFE‐rich oils in equimolar AzoTAB‐SDS, under the application of ultraviolet light, with inset micrographs of a decane‐rich sessile drop before measurements and after application of ultraviolet light. Error bars represent the standard deviation of n > 5 experimental measurements.

Under ambient light conditions, the surfactant assembly predominantly resided in the aqueous phase. However, upon UV irradiation, significant partitioning into the oil phase was observed, as evidenced by the increased visible absorbance of AzoTAB (**Figure** **2**a). This partitioning was further corroborated by NMR analysis of both phases, confirming the shift in surfactant distribution (Figure S11, Supporting Information).

To measure the uptake of surfactant assembly into the oil phase we next analyzed the resulting volume changes of single‐phase emulsion droplets placed next to the surfactant assemblies. We prepared spherical droplets composed of a homogeneous mixture of two oils (octane:HFE7500) in equimolar AzoTAB:SDS and directly observe droplet volume changes on the application of either ultraviolet or blue light (Figure 2b and Video S3, Supporting Information).

The mixture of two oils was homogenous and the fluorocarbon was employed solely to ensure gravitational settling of droplets inside the aqueous continuous phases. Upon application of ultraviolet light, the droplets assume a resting state at an elevated volume, while blue light reduces their volume to a lower resting state (Figure 2c). This indicates that the coacervate formed by *cis*‐AzoTAB‐SDS in ultraviolet light preferentially partitions within the oil droplets, temporarily raising the volume of the respective droplet phase. Vice versa, when blue light is applied and the crystalline *trans*‐AzoTAB‐SDS assembly is formed, rapid precipitation from the aqueous continuous phase results in a shift in the surfactant partitioning equilibrium toward the aqueous phase, consequently lowering the volume of the droplets (Figure 2c). When droplets were dispersed in aqueous phases containing near‐equimolar mixtures of AzoTAB and SDS partial de‐emulsification is observed as attributed to the precipitation and removal of surfactants.

Interestingly, when tracking the volume, we observed a peak in volume accompanying the phase transition and associated shift in partitioning equilibrium (Figure 2c; Figure S13, Supporting Information). This peak volume is proportional to the initial droplet volume, as expected due to Ostwald ripening (Figure 2d). The pronounced peak suggests a transient and significant change in interfacial tension that is associated with a shift in the steady‐state surfactant distribution. We can attribute this to the phase transition of the surfactant assembly from the aqueous phase into the oil phase, temporarily broadening the interface and altering the droplet environment. The transient formation of a surfactant‐rich interphase can result in significantly lowered interfacial tensions.^[^ ^36^, ^37^ ^]^ Consequently, we observe a second peak actuation as the partitioning equilibrium shifts back toward the aqueous phase. As indicator of a change in interfacial tension due to partitioning of the surfactant assembly into the droplets, we determined the upper critical solution temperature of a biphasic oil mixture. The addition of a tertiary component to droplets can influence the upper critical solution temperature through induced or reduced miscibility of the constituent phases (Figure 2e; Figure S12 and Table S2, Supporting Information).^[^ ^38^ ^]^ When droplets in AzoTAB‐SDS are placed inside a temperature‐controlled stage, the upper critical solution temperature is observed to have raised between 3 °C and 5 °C depending on the application of ultraviolet or blue light. However, measurement of this interfacial tension is subdued due to two factors, first is a variation of the *cis‐trans* equilibrium of AzoTAB, due to preferential isomerization toward the *trans* isomer at elevated temperature, and second is the temperature‐dependent thermotropic nature of AzoTAB‐SDS and subsequent equilibrium with monomeric units. To observe the changing upper critical solution temperature as a macro‐scale effect, the partitioning of AzoTAB‐SDS can trigger phase separation or mixing for droplets that present an upper critical solution temperature near room temperature (Figure S15, Supporting Information).

Pendant drop interfacial tension measurements of decane‐rich or HFE‐rich single‐phase droplets in pure solutions of SDS yielded values of 18–25 mN m^−1^ in SDS, and 6–7 mN m^−1^ in AzoTAB, respectively (Figure 2f). When measured in solutions containing both AzoTAB and SDS, a substantial drop in interfacial tension was observed, while steady pendant droplets were not achievable when switching in between ultraviolet or blue light illumination. The latter indicates a very low transient interfacial tension and non‐equilibrium behavior of the pendant droplets, as now expected from the dynamic effects the phase‐transfer of the surfactant assemblies have on the system.

To measure the low interfacial tensions, we employed a liquid sessile drop method allowing measurement of droplets large enough to deform out of sphericity.^[^ ^39^, ^40^, ^41^ ^]^ For decane‐rich as well as HFE7500‐rich droplets in aqueous solutions of AzoTAB‐SDS, large droplets deform out of sphericity within seconds under UV illumination (Figure 2g and Video S4, Supporting Information). The measurements reveal a minimum interfacial tension on the order of 0.1–0.5 mN m^−1^ (Figure 2g). From pure mixtures of either SDS or AzoTAB (Figure 2f), the combined effects of partitioning and surfactant equilibria therefore lowered the effective decane‐water interfacial tension by 250–989‐fold, from 18 mN m^−1^ in SDS and 5 mN m^−1^ in AzoTAB to 0.019 mN m^−1^ in a mixture of both. For the fluorocarbon HFE‐7500, a similar decrease is observed, with changes in interfacial tension in the mixture by a factor of 22, from 7 to 0.3 mN m^−1^. Notably, in these experiments, the measured sessile droplets have a far larger volume on the order of 10^9^ µm3, with respect to the emulsion droplets which are on the order of 10^4^ µm3. Consequently, the sessile droplets compartmentalize larger amounts of the surfactants. This difference is also reflected in the time‐scale of the experiment, which increased to 10 s (Figure 2g).

To better understand the transient variations in interfacial tension we next exposed Janus emulsion droplets to the SDS and AzoTAB containing continuous phase. Typically, variations in surfactant efficacy and the resulting changes in interfacial tension lead only to qualitative changes in single‐phase emulsion droplets, such as droplet destruction or coalescence. In Janus droplets, variations in morphology can be evoked in response to dynamic variations in surfactant effectiveness, while the overall droplet system remains intact.^[^ ^42^ ^]^ This is due to the use of a second inert surfactant that maintains the stability of the Janus droplets during stimulation. Janus droplets thereby serve as a messenger tool to report and quantify transient changes in interfacial tension in real time.^[^ ^43^, ^44^ ^]^

**Figure 3:**
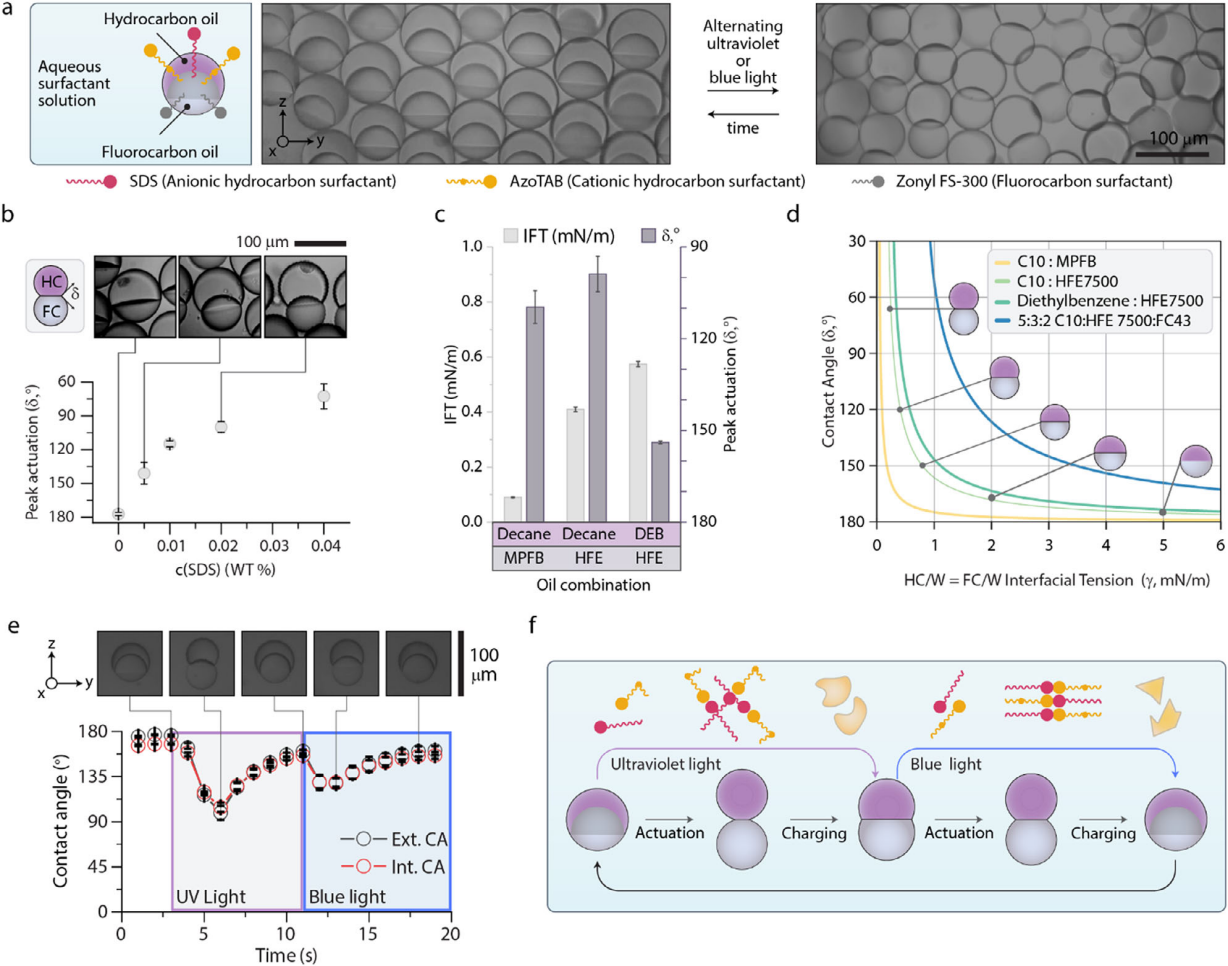
Complex emulsions actuate non‐spherically in solutions of AzoTAB and SDS. a) Sideview optical micrographs of Janus emulsion droplets composed of decane and HFE7500 in solutions of AzoTAB, sodium dodecyl sulfate, and Zonyl FS‐300, as outlined in the schematic, actuating in response to alternating blue and ultraviolet light irradiation. b) Peak non‐spherical actuation contact angles (δ) for droplets in 0.15 WT% AzoTAB, and increasing concentrations of SDS. c) Oil–oil interfacial tension values and peak actuation contact angles (δ) in uniform concentrations of AzoTAB and SDS, for droplets of different oil compositions. d) Calculated hydrocarbon‐water and fluorocarbon‐water interfacial tensions versus external contact angles (δ) for varying hydrocarbon‐fluorocarbon interfacial tensions (from c) with inlaid schemes of respective non‐spherical droplet morphologies. e) Time‐resolved contact angle (δ, θ) measurements of droplets in equimolar AzoTAB‐SDS on the application of ultraviolet and blue light, with inset micrographs of 1 droplet at the noted locations f) On the application of ultraviolet light, SDS‐AzoTAB precipitate takes a coacervate form, which partitions into the droplet, generating a local low interfacial tension gradient and raising the internal interfacial tension, actuating the droplet. On application of blue light, coacervated AzoTAB‐SDS crystallizes, preferring the aqueous phase, and on exit of the droplet generates another local interfacial tension gradient. Error bars represent the standard deviation of n > 5 experimental measurements.

When spherical Janus emulsions are placed in solutions of AzoTAB with a small amount of the hydrocarbon surfactant sodium dodecyl sulfate (SDS), we observe actuation from spherical to non‐spherical shapes with the application of successive ultraviolet or blue light (**Figure** **3**a). As the concentration balance of AzoTAB and SDS is changed, the peak actuation of the droplet can be tuned (Figure 3b), until droplets separate at less than one‐quarter of the CMC of SDS at 0.04 WT % SDS (CMC = ≈0.215 WT %, 8.4 mM), with 0.15 WT % AzoTAB. In uniform concentrations of AzoTAB and SDS, droplets composed of different oil combinations, including decane:methoxyperfluorobutane, decane:HFE7500, and diethylbenzene:HFE7500 all present spherical to non‐spherical transitions in the same time interval (Figure 3c), indicating the effect is not attributable to specific interactions between the oils. The decane (hydrocarbon‐rich phase) – HFE7500 (fluorocarbon‐rich phase) interfacial tension is measured to be 0.4 mN m^−1^ (Figure 3c).

Negating precipitation‐induced surfactant depletion, Janus emulsion droplets in solutions of AzoTAB‐SDS remain stable due to the presence of the additional stabilizing nonionic fluorocarbon surfactant Zonyl (0.25 WT %), enabling in situ observation of AzoTAB‐SDS dynamics. Starting with a system illuminated with non‐interacting red light (λ = 660nm), when ultraviolet light (λ = 365nm) is applied, droplet actuation is observed within seconds, settling at a non‐spherical snowman‐shaped morphology (Figure 3a). Conversely, when blue light (l = 470 nm) is applied, we observe a second peak actuation on the order of 2 s followed by settling of the droplets in a near‐spherical morphology under continuous blue light illumination. Theoretical considerations reveal that, when assuming a constant hydrocarbon‐fluorocarbon interfacial tension (Table S4, Supporting Information), the observed non‐spherical snowman‐type morphology actuations require the droplet external oil‐water interfacial tensions to also be reduced by an order of magnitude to values on the order of 0.4 mN m^−1^ (from ≈5 mN m^−1^), as determined from the force equilibrium of interfacial tensions given by the Neumann triangle (Figure 3d).^[^ ^45^, ^46^ ^]^

We also prepared Cerberus emulsion droplets comprising three internal oil phases (hydrocarbon, fluorocarbon, and silicon‐oil). Upon placing the Cerberus droplets in 5 mM AzoTAB:SDS, we observe that only the upper hydrocarbon phase breaks out of sphericity. Thus, while the effect on the internal oil‐oil interfacial tensions is not sufficient to fully actuate all droplet phases, the equalization of interfacial tensions follows the local partitioning of AzoTAB‐SDS into the hydrocarbon‐rich phase of the droplets.

Based on the characterization of solubility‐induced partitioning into the droplets, as well as the partitioning equilibria induced gradients in interfacial tension (Figure 3e), a comprehensive description of the two‐stroke charging‐actuation cycle of the system can be proposed (Figure 3f). Starting from ambient conditions, where AzoTAB‐SDS precipitate and coacervate as well as monomeric surfactants (AzoTAB and SDS) are in equilibrium, a UV light‐induced switching to the *cis‐*AzoTAB‐SDS assembly is followed by a preferred solubility of the assembly into one droplet phase. This induces local concentration gradients of surfactants adjacent to the droplet interface, which lower interfacial tension. The partitioning of the supramolecular assembly and the equilibria monomeric surfactant is the driving force to lower the interfacial tensions, even as the overall concentration of surfactant in the system is below the critical micelle concentration of one component. Therefore, the spherical to non‐spherical actuation is the observation of a dynamic and collaborative effect, attributed to a transient minima interfacial tension mediated by the transition of AzoTAB:SDS from water‐soluble to oil‐soluble states. The measurement of peak actuation during phase transfer of AzoTAB‐SDS into and out of the droplet, combined with the measured changes in internal HC‐FC interfacial tensions, and HC‐W and FC‐W interfacial tensions is indicative of this collaboration.

The linear actuation of complex emulsion droplets is thus attributed to the simultaneous lowering of the external interfacial tension, and a raise in internal interfacial tension in tandem. Due to the equilibrium between both coacervate, crystal, and monomeric states of the surfactants, on the transition from water to oil, a local elevated concentration of surfactant induces transient lowered hydrocarbon/fluorocarbon‐water interfacial tension. Simultaneously, the presence of the *cis* AzoTAB‐SDS within the oil phase of the emulsion droplet allows for these effects to induce a spherical to non‐spherical transition: lowering the hydrocarbon‐water and fluorocarbon‐water interfacial tensions, and raising the hydrocarbon‐fluorocarbon interfacial tension.

In turn, on the application of blue light, *trans* AzoTAB‐SDS preferably precipitates as a solid crystalline phase from water, depleting the monomeric surfactant in water, subsequently driving surfactant partitioning into the water phase. During surfactant transfer into the water phase a locally elevated concentration of surfactant is present adjacent to the oil‐water interface, again significantly lowering the hydrocarbon‐fluorocarbon interfacial tension, before Janus droplets come to rest at a near‐spherical state once equilibrium state is reached.

**Figure 4:**
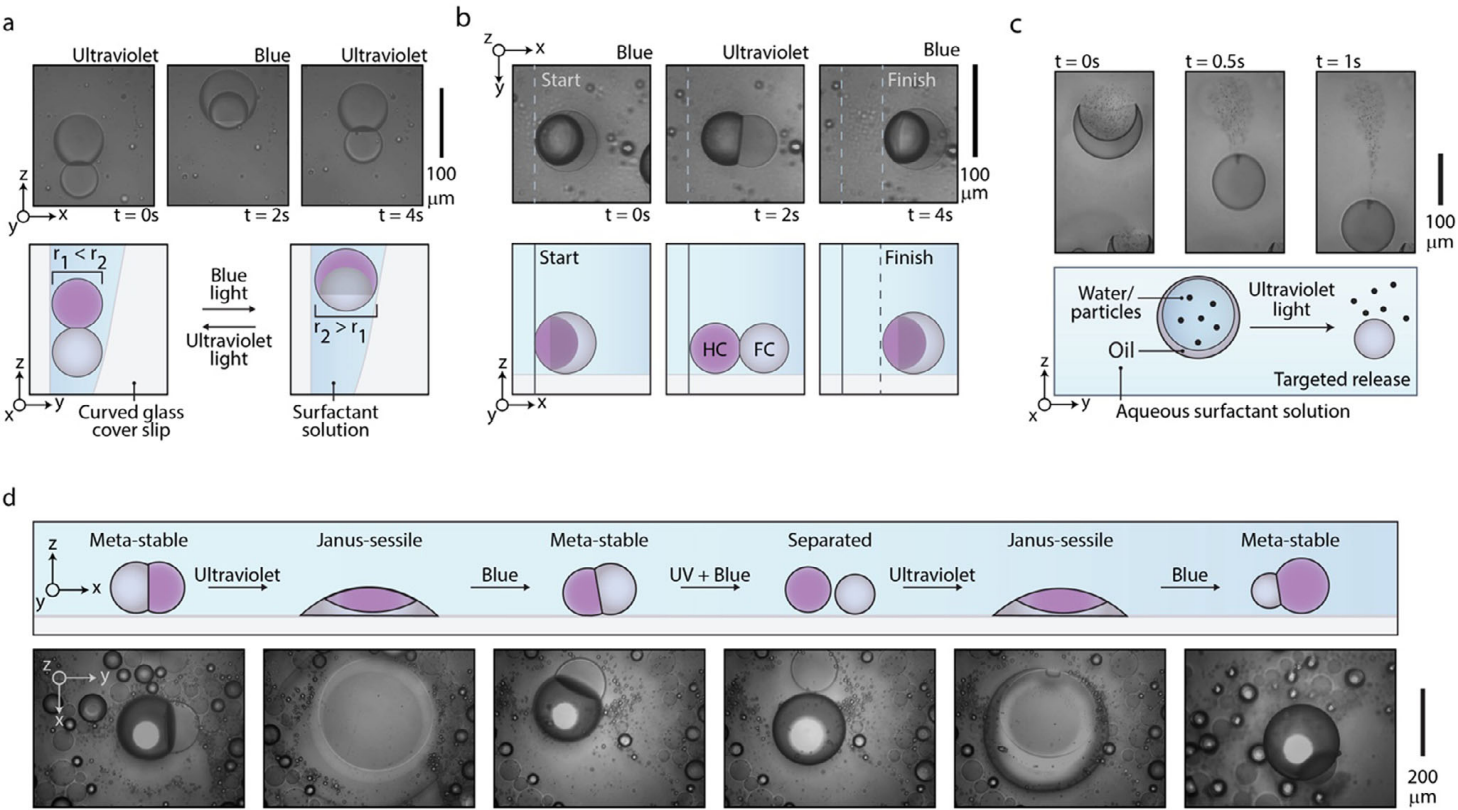
Droplets act with physicality using the collaborative interfacial effects of AzoTAB‐SDS. a) Complex emulsion droplets in an AzoTAB‐SDS and Zonyl solution within an angled sample holder, which physically push against the holder to move vertically. b) Complex droplets that have two phase with matching densities sit on their side with respect to gravity, by repeatedly actuating the droplets between spherical and non‐spherical conformations, the droplets can translate using physical motion. c) Time‐resolved micrographs of water‐in‐oil‐in‐water double emulsion droplets can be selectively destabilized by inducing phase‐transfer of AzoTAB‐SDS into the droplet, ultimately releasing the aqueous cargo (tracer particles) into the environment. d) Time‐resolved micrographs of several behaviors mediated by changing the balance of blue and ultraviolet light for droplets which are stabilized in a solution of AzoTAB‐SDS with Zonyl below the CMC, where droplets are 1. Meta‐stable, 2. Destabilized with pure ultraviolet light, 3. Semi‐stabilized with blue light, 4. split with an equal balance of blue and ultraviolet light, 5. Re‐joined with pure ultraviolet light, and finally stabilized with pure blue light.

To visualize significant surface aspect ratio variations of biphasic Janus droplets evoked by switchable surfactant assemblies, we placed droplets in an angled cell, where the force generated by the changing droplet diameters pushes against gravity (Discussion S3 and Table S2 and Video S5, Supporting Information). When the radius of the droplet is changed due to the linear actuation, the droplet moves vertically, pushing against the glass substrate (**Figure** **4**a).

Droplets usually rest aligned with gravity with the denser droplet phase at the bottom, which can be upended by modifying the density balance of droplet fluids. Janus droplets, which present two phases with an equal density greater than water (ρ = 1.52 g mL^−1^; diethylphthalate, dibromomethane, and HFE7500) sit on their side. By quickly actuating the droplets on the second timescale, we realize a caterpillar‐like motion, with 52 micrometers of traversed distance in 4 strokes over 10 s of light actuation (droplet radius: ≈50 µm; Figure 4b and Video S6, Supporting Information).

Next, the same principles of increasing the interfacial tension at the internal droplet interface via partitioning of coacervated AzoTAB‐SDS can be extended to a single‐phase droplet system comprised of hydrocarbon and fluorocarbon oils in their mixed state below their respective UCST (diethyl phthalate, dibromomethane, HFE7200). A light‐controlled partitioning‐induced de‐mixing thereby provides as reversible method for chemical separation of the hydrocarbon and fluorocarbon oils (Figure S12, Supporting Information). This separation is directed, and limited by light, and is therefore selected by the placement of the light spot, or can be generalized for Janus emulsions placed in aqueous solutions of large concentrations of AOT (Figure S12, Supporting Information).

The temporarily generated ultra‐low interfacial tension during phase‐partitioning of AzoTAB‐SDS with ultraviolet light can also enable selective destabilization of water‐in‐oil‐in‐water double emulsions, where the encapsulant is released into the aqueous continuous phase, while the low‐concentration AzoTAB‐SDS is selectively crystallized from the water phase and out of the separated Janus or double‐emulsions (Figure 4c and Video S7, Supporting Information).

Light‐controlled dissipative supramolecular surfactant assemblies can be employed to control both the droplet actuation and stability. In a single continuous experiment, where we use droplets in solutions of AzoTAB and SDS, where SDS and AzoTAB is below their CMC in the *cis* conformation, and AzoTAB is above its CMC in the *trans* conformation, several tactile droplet behaviors can be demonstrated. With actuation on the second‐time scale, a stable droplet can be destabilized against the substrate upon application of ultraviolet light (Figure 4d and Video S8, Supporting Information). By controlling the application and balance of ultraviolet and blue light, enough free surfactant can be directed in and around the sessile droplet to partially re‐stabilize the emulsion droplet with more blue than ultraviolet light. By raising the proportion of ultraviolet light, the droplet phases are then split into two separate droplets, which will remain separated under confinement (Figure S15, Supporting Information). By applying pure ultraviolet light, a sessile droplet is once again generated, which collects fellow broken emulsion droplets. When the balance of light becomes once again blue‐dominated, the droplet reverts back to a meta‐stable Janus droplet (Figure 4d), having now collected fellow broken emulsion droplets and increasing in volume from 2.1 10^6^ µm3 to 7.9 10^6^ µm3. By reversibly stabilizing and de‐stabilizing meta‐stable droplets in 1 s, the droplets can crawl due to the transition between sessile and meta‐stable states (Figure 4d and Video S8, Supporting Information).

## 3 Conclusion

We report a light‐controlled, reversible crystal‐to‐coacervate transition of a countercharged surfactant assembly as a novel stimuli‐triggered mechanism for inducing order‐of‐magnitude changes in interfacial tension within seconds. The effect is driven by a photoinduced solid‐to‐liquid phase transition of an ionic supramolecular assembly formed between anionic sodium dodecyl sulfate (SDS) and cationic azobenzene‐based (AzoTAB) surfactants. Accompanying this phase transition, a reversible partitioning of surfactant assemblies between the oil and aqueous phases of an emulsion transiently reduces the effective oil‐water interfacial tension by up to 989‐fold within seconds, as compared to interfaces stabilized by either SDS or AzoTAB.

These findings introduce a fundamentally new mechanism for chemo‐mechanical transduction in purely liquid systems, enabling rapid, multimodal shape transformations and actuation of emulsion droplets within seconds. The integration of a spring‐like charging and latch‐controlled release mechanism provides a level of force amplification far beyond conventional stimuli‐responsive surfactant systems, unlocking new possibilities for transient structuring and shape programming of all‐liquid matter. In addition to actuating single‐phase droplets out of sphericity, we demonstrate actuation, splitting, destabilization, and recombination of double and Janus emulsion droplets, generating tactile forces proportional to the minimum interfacial tension in the system and the droplet size, on the micronewton scale in 1 s. The light‐powered phase transition of supramolecular surfactant assemblies operates between two reversible and controllable energetic minima. Such chemo‐mechanical systems, leveraging multiple recoverable and object‐specific energy states at low concentrations, hold broad potential for applications in chemical purification, phase separation, and oil recovery. Imparting liquid objects with second‐scale mechanical feedback has broad implications in areas of micromanipulation and the realization of life‐like soft matter, where behaviors such as crawling, sessile crawling, and targeted release from liquid capsules can find abundant application.

## Conflict of Interest

The authors declare no conflict of interest.

## Supporting information



Supporting Information

Supplemental Video 1

Supplemental Video 2

Supplemental Video 3

Supplemental Video 4

Supplemental Video 5

Supplemental Video 6

Supplemental Video 7

Supplemental Video 8

Supplemental Video 9

## Data Availability

The data that support the findings of this study are available in the supplementary material of this article.
