# Supplementary material for: Reversible Phase Transitions of Anionic and Cationic Surfactant Mixtures Drive Shape Morphing Droplets
Source: Adv Mater. 2025 Jun 26;37(37):2506100. doi: 10.1002/adma.202506100 (PMC12447037; doi:10.1002/adma.202506100)
Supplement: Supplementary file 1 — Supporting Information [file ADMA-37-2506100-s006.docx]

Reversible phase transitions of anionic and cationic surfactant mixtures drive shape morphing droplets

Bradley D. Frank,^1^ Pilar Romero,^2^ Alberto Concellón,^2^ and Lukas Zeininger^1,^*

^1^Department of Colloid Chemistry, Max Planck Institute of Colloids and Interfaces, Am Mühlenberg 1, 14476 Potsdam, Germany; lukas.zeininger@mpikg.mpg.de

^2^Instituto de Nanociencia y Materiales de Aragón (INMA), CSIC-Universidad de Zaragoza, ES-50009 Zaragoza, Spain

Supplementary Information

[Materials 3](#_Toc200634840)

[Methods 3](#_Toc200634841)

[Preparation of Janus emulsion droplets 3](#_Toc200634842)

[Preparation of water-in-oil-in-water double emulsion droplets 4](#_Toc200634843)

[Synthesis of C4-AzoTAB 4](#_Toc200634844)

[Molecular characterization of AzoTAB-SDS via NMR 4](#_Toc200634845)

[Characterization of emulsion droplets 4](#_Toc200634846)

[Pendant drop tensiometry 5](#_Toc200634847)

[Spinning drop tensiometry 5](#_Toc200634848)

[Liquid sessile droplet tensiometry 5](#_Toc200634849)

[Density measurements 6](#_Toc200634850)

[Upper Critical Solution Temperature 6](#_Toc200634851)

[Supplementary Discussion 7](#_Toc200634852)

[Supplementary Discussion 1. NMR studies of AzoTAB-SDS 7](#_Toc200634853)

[Supplementary Discussion 2. Measurement of oil-water interfacial tensions with AzoTAB-SDS 8](#_Toc200634854)

[Supplementary Discussion 3. Determination of linear force generated by spherical-non-spherical transitions 9](#_Toc200634855)

[Supplementary Tables 10](#_Toc200634856)

[Supplementary Table S1. Diffusion coefficients calculated by DOSY NMR experiments. 10](#_Toc200634857)

[Supplementary Table S2. Densimetry measurements of hydrocarbon and fluorocarbon oils used in this study. 11](#_Toc200634858)

[Supplementary Table S3: UCST For oil combinations used in the paper 12](#_Toc200634859)

[Supplementary Table S4: Interfacial tensions of hydrocarbon-fluorocarbon oil mixtures 13](#_Toc200634860)

[Supplementary Figures 14](#_Toc200634861)

[Supporting Videos 32](#_Toc200634862)

[Supporting Video 1 32](#_Toc200634863)

[Supporting Video 2 32](#_Toc200634864)

[Supporting Video 3 32](#_Toc200634865)

[Supporting Video 4 32](#_Toc200634866)

[Supporting Video 5 32](#_Toc200634867)

[Supporting Video 6 32](#_Toc200634868)

[Supporting Video 7 32](#_Toc200634869)

[Supporting Video 8 33](#_Toc200634870)

[Supporting Video 9 33](#_Toc200634871)

[References 34](#_Toc200634872)

# Materials

All chemicals were used as received without further purification. n-Decane (Sigma, 99%), HFE-7500 (ABCR, 99%), HFE 7200 (ABCR, 99%), Diethyl benzene, diethyl phthalate (Sigma, 99%), dibromomethane (Sigma, 99%), methoxyperfluorobutane (ABCR 99%), sodium dodecyl sulfate (Sigma 99%), Krytox FSH 157 (ABCR), Dioctyl sulfosuccinate sodium salt (Sigma, 97%), Hexadecyltrimethylammonium bromide (Sigma, 98%), 1 micrometer latex spheres (Polybead), Silicone oil (50 Cst, Sigma).

# Methods

## Preparation of Janus emulsion droplets

Janus emulsion droplets were prepared either with batch mixing thermal phase separation technique, or with room temperature microfluidics, generating droplets between 25 and 100 micrometers in radius.^1^ For batch mixing, oil combinations that display an upper critical solution temperature below the boiling point of either liquid are chosen (Table S1). These constituent oils in equal volume fraction are placed in a vial and heated. In a second vial, an accompanying surfactant solution above the UCST temperature by a few degrees. A pipette tip is inserted into the heated, mixed oils, and 20 microliters of mixed hydrocarbon/fluorocarbon oil are pipetted into the warm surfactant solution, and the surfactant solution immediately vortex mixed for 10 seconds at 2500 RPM. Experiments are carried out after cooling to room temperature. For room temperature microfluidics, a 5-way hydrophilic glass chip from Dolomite is used, with two surfactant channels, and one inlet for each oil, in conjunction with Fluigent EZ series pumps. All droplets were used within 48 hours of generation and are meta-stable, on the order of three months in a refrigerator while susceptible destabilization mechanisms, such as solubilization into the continuous phase. Droplets were characterized with side view optical microscopy, with a custom-built microscope setup (Supporting Figure S13), and the morphology was tuned with a change in balance of interfacial tensions controlled by hydrocarbon surfactants (e.g., sodium dodecyl sulfate) and fluorocarbon surfactants (Zonyl FS-300).

## Preparation of water-in-oil-in-water double emulsion droplets

Double emulsions were prepared with a two-step vortex approach introduced by Wang and coworkers,^2^ of which the protocol was used without modification and is repeated here in brief: In a 15 mL centrifuge tube (Tube **1**), 1 mL of a 1 WT % solution of Krytox FSH 157 in HFE-7500 was placed, before 200 microliters of dilute 1 micrometer polystyrene sphere solution was pipetted into it. Tube **1** was vortex mixed for 30 seconds at 2500 RPM, and left to rest for 5 minutes. Using a plastic pipette, some amount of the creamed oil-in-water emulsion was drawn into the pipette, and the pipette again allowed to rest, before excess oil which had settled in the pipette tip was removed. This fraction was then pipetted into Tube **2** which contained a 1 WT % solution of sodium dodecyl sulfate in water. This was then vortexed for 30 seconds at 2500 RPM, and contained a polydisperse water-in-fluorocarbon-in-water double emulsion. For experiments within the paper, these droplets were phase-transferred into a solution of equimolar AzoTAB-SDS at a total concentration of 2.5 mmol, and were found to collapse, releasing the internal water phase and retaining the oil shell as an emulsion droplet on the application of ultraviolet light.

## Synthesis of C4-AzoTAB

The synthesis of C4-AzoTAB has been previously reported.^3^ The C4-AzoTAB used in this work was synthesized following an unmodified literature-reported protocol.^4^

## Molecular characterization of AzoTAB-SDS via NMR

NMR experiments were carried out on Bruker Avance Neo spectrometer operating at 500 MHz for ^1^H using standard pulse sequences. Chemical shifts are given in ppm relative to TMS and the residual solvent peak was used as internal reference.

## Characterization of emulsion droplets

Emulsion droplets within the paper were characterized using various computation methods. For the measurement of emulsion droplets, the contact angle with reference to the fluorocarbon phase was used to characterize the internal geometry of the emulsion droplets. Measurements of droplet contact angle were corrected due to refractive lensing.^5^ For the external contact angle, the angle between the tangent of either droplet phase was used to describe the external contact angle, as previously described by Guzowski and coworkers.^6^ For the measurement of volume dependance with time, droplets were tracked using a modified version of the particle tracking software by Crocker and Grier,^7^ for use with droplets, time-dependent radius and location data were used to estimate the volume of droplets over time. These droplets were observed from above, and from the side to verify that the change in radius was solely due to a changing volume and not a change in sphericity. Time-dependent volume data was smoothed with a gaussian filter with a width of 10 frames.

## Pendant drop tensiometry

Measurements of interfacial tension with pendant drop tensiometry was undertaken in a custom-built in-dark setup to correct for lighting, where a large diffuse light spot (660 nm) was placed behind the cuvette, and ultraviolet or blue light was delivered perpendicularly to the cuvette. Droplets were generated with a syringe pump to keep ambient light from the sample, due to the presence of ultraviolet and blue light in the visible spectrum. Droplet images were measured with OpenDrop,^8^ and dynamic interfacial tension was fit to find the final interfacial tension according to a previously reported procedure.^1^

## Spinning drop tensiometry

Spinning drop tensiometry was used to measure oil-oil interfacial tensions, using a Krüss SITE100 with the small droplet volume attachment. After cleaning, the capillary was filled with the heavier fluorocarbon phase which was pre-mixed and separated at room temperature. The capillary was controlled to room temperature (23°C, as with all density and interfacial tension measurements in this work). This phase was used to measure the calibration rod for the camera before the capillary was sealed. After spinning to 1000 RPM, 1 microliter of the internal hydrocarbon phase was injected into the capillary tube and allowed to equilibrate. Once a stable droplet was produced, measurements of interfacial tension were made at 5000 RPM

## Liquid sessile droplet tensiometry

Measurements of interfacial tension below 1 mN/m were undertaken with liquid sessile droplet tensiometry.^9, 10^ Droplets with measured densities were placed in the less-dense phase, and the deformation of the droplet under the influence of gravity was utilized to determine the interfacial tension within the system. Droplet images were analyzed with a modified version of the code by Dufresne and coworkers,^11^ to monitor the volume of the droplets, and ensure that the droplet width did not reach the width of the sample container. For light responsive experiments, light was controlled with successive and timed blue and ultraviolet collimated light to the sample, illuminated with solely red light for imaging.

## Density measurements

For density experiments, 3 mL of one hydrocarbon and one fluorocarbon oil pairing was mixed and heated above its upper critical solution temperature and thoroughly mixed before left to cool on the bench for 24 hours. Once thoroughly phase separated at room temperature, a syringe was used to uptake 1.8mL of first the fluorocarbon-rich, and then the hydrocarbon rich phase. One phase was inserted into a cleaned, dried, and temperature controlled Anton Paar DMA 5000 M Densimeter. The densimeter was checked to air density (per local pressure and temperature) before every experiment. Subsequent oil was inserted for 5 measurements. The device was cleaned with ethanol, acetone, and dried between experiments and verified to local air density.

## Upper Critical Solution Temperature

To measure the upper critical solution temperature (UCST) of oil droplet combinations, droplets were first prepared in a solution of Zonyl FS-300 at 0.25 WT %, above the critical micelle concentration. These droplets were placed in a heating stage, namely a Linkam TP-94 and Linkam LNP. To measure and verify the UCST of the droplets, droplets were heated well above the experimental upper critical solution temperature used for preparation, and cooled at 1°C per minute to find an approximate UCST. Then, the droplets were heated (1°C per minute) and cooled (0.1°C per minute) to measure the upper critical solution temperature, taken as the temperature at which turbidity is observed in droplets which begin to de-mix.

# Supplementary Discussion

## Supplementary Discussion 1. NMR studies of AzoTAB-SDS

Utilizing diffusion ordered spectroscopy (DOSY) NMR, it is evident that *trans* AzoTAB forms micelles that interact with water molecules, incorporating monomeric SDS molecules. In contrast, the *cis* isomer forming a complex with SDS presents a diffusion coefficient similar to that of monomeric species. **Table S1** compiles the diffusion coefficients of the complexes in a 10:1 ratio and their components, while **Supplementary Figure S4** displays DOSY spectra for the 10:1 *trans* and *cis* complexes of AzoTAB at 5mM.

To gain insight into the incorporation of the SDS surfactant into the *trans* AzoTAB micelle, ^1^H-^1^H nuclear Overhauser effect spectroscopy (NOESY) NMR experiments were conducted with varying mixing times. Cross-peaks are clearly discernible between the protons of the SDS chain and the aromatic protons of AzoTAB, indicating the close proximity of these two molecules as a consequence of SDS interdigitation within AzoTAB micelles (**Supplementary Figure S5**).

To understand the photoresponsive supramolecular assembly of cationic AzoTAB and anionic SDS surfactants in the aqueous phase, a molecular-level investigation was conducted utilizing NMR. Initial titration experiments in D_2_O were performed by gradually introducing SDS to AzoTAB solutions in both its trans and cis states (i.e., before and after irradiation with UV light) (**Supplementary Figures S6** and S**7**).

## Supplementary Discussion 2. Measurement of oil-water interfacial tensions with AzoTAB-SDS

A variety of techniques were employed to measure the interfacial tension of AzoTAB-SDS in this manuscript. Pendant drop is an effective technique for interfacial tensions above 1 mN/m, and it was quickly found that in any solution containing AzoTAB and SDS at concentrations and equilibria used in this paper, stable droplets could not be formed. To measure smaller interfacial equilibria, a spinning drop tensiometer was employed to directly measure the oil-oil interfacial tension. While spinning drop tensiometer is useful to measure oil-oil interfacial tensions, the setup is limited by two key features: one is the dependance on refractive index, and two is the limitation of possible phases within the device. For any partitioning-induced effect, the refractive index of either phase and therefore the apparent size of the droplet will change, hindering measurement. Furthermore, the larger density phase must remain on the outside of the spinning drop, which would mean flipping the volume balance of water, hydrocarbon, and fluorocarbon, which is critical to the magnitude of the effect.

The precipitation, and the crystal-coacervate transition of AzoTAB-SDS is challenging to measure with traditional means due to the speed of the effect. To measure the hydrocarbon-fluorocarbon interfacial tension at its lowest point, or at the peak actuation of a hydrocarbon-fluorocarbon droplet (Manuscript Figure 3a), the possibility of pre-loading one or both oil phases is unfortunately out of question for precise measurement not only because of the transient nature of this interaction, but also the scale of required volume and following equilibria. To answer this problem, the liquid sessile droplet method, even with respect to three primary sources of error: first is the unknown density of the measured droplet, the volume-dependance of the droplet for measurement, and the changing lighting conditions to AzoTAB-SDS due to Beer-Lambert law. This also serves to explain the changing timescale of droplet actuation when compared to large sessile droplets, as millimeter-scale droplets are placed in a larger millimeter scale cuvette, compared to micron-scale Janus droplets which are placed and imaged in a vertical quasi Hale-Shaw cell. Ultimately, we consider the measurements of interfacial tension in this system as a minimum and on the order, which demonstrate both the external and internal effects of changing equilibria, partitioning, and interfacial tension gradients.

## Supplementary Discussion 3. Determination of linear force generated by spherical-non-spherical transitions

Considering the measured interfacial tension in the system as an absolute minimum with consideration for the sources of error outlined in Supporting Discussion 2, the minimum force (and power) generated by the droplets can be estimated. The volume of the Janus droplet, **V_droplet_**, is proportional to either droplet phase **V1** and **V2** where,

$$V_{droplet}=V_{1}+V_{2}$$

and the unexpended length, **l_1_**, of the spherical Janus droplet is proportional to the volume.

$$l_{1}=2\sqrt[3]{\frac{3}{4} {\pi V}_{droplet}}$$

For any spherical droplet with a non-spherical actuation, there is a maximum length of extension (Supporting Figure S17), **l_2_**, before the droplet phases are separated, proportional to the volume of the droplet **V_droplet_** and either droplet phase **V_1_** and **V_2_** where,

$$l_{2}= 2\sqrt[3]{\frac{3}{4} {\pi V}_{1}}+ 2\sqrt[3]{\frac{3}{4} {\pi V}_{2}}$$

assuming that the maximum extension of the droplet is the point just before the two droplet phases separate. As interfacial tension, gamma (N/m), is defined as,

$$\gamma=\frac{F}{2l}$$

Where **F** is force in newtons, and **L** is the length of extension in meters, a known interfacial tension in the system can be used to derive the force being exerted for any resisted extended length, this results in the maximum force of the droplet being proportional to both interfacial tension and extension length.

$$F=2 \gamma l_{2}$$

As the force is proportional to the resisted length, and the length within the system is proportional to droplet volume, larger droplets will have larger actuation forces, which is also tunable by the minimum interfacial tension being overcome in the system (Supporting Figure S17).

# Supplementary Tables

## Supplementary Table S1. Diffusion coefficients calculated by DOSY NMR experiments.

|  | **D_molecule_ (m^2^s^-1^)** | **D_water_ (m^2^s^-1^)** |
| --- | --- | --- |
| SDS, 5mM | 4.21 x 10^-10^ | 1.76 x 10^-9^ |
| (cis) AzoTAB, 5mM | 3.25 x 10^-10^ | 1.76 x 10^-9^ |
| (trans) AzoTAB, 1.2mM | 3.71 x 10^-10^ | 1.71 x 10^-9^ |
| (trans) AzoTAB, 5mM | 1.04 x 10^-10^ | 1.76 x 10^-9^ |
| (cis) AzoTAB-SDS (10:1) | 3.12 x 10^-10^ | 1.73 x 10^-9^ |
| (trans) AzoTAB-SDS (10:1) | 6.94 x 10^-11^ (AzoTAB)  1.82 x 10^-10^ (SDS) | 1.73 x 10^-9^ |

## Supplementary Table S2. Densimetry measurements of hydrocarbon and fluorocarbon oils used in this study.

| Oil combination | HC Pure  ρ (g/mL) | FC Pure ρ (g/mL) | HC rich ρ (g/mL) | ρ HC err | FC rich ρ (g/mL) | ρ HC err |
| --- | --- | --- | --- | --- | --- | --- |
| Decane : Methoxyperfluorobutane | 0.730 | 1.520 | 0.9129 | 6.34E-06 | 1.3253 | 2.09E-05 |
| Diethylbenzene : HFE7500 | 0.870 | 1.614 | 0.9496 | 4.47E-05 | 1.4598 | 8.71E-03 |
| Decane : HFE7500 | 0.730 | 1.614 | 0.8530 | 2.01E-05 | 1.4578 | 1.08E-05 |
| 10:9:1 Decane : HFE7500 : FC43 | 0.730 | 1.614:  1.860 | 0.8494 | 4.75E-04 | 1.4868 | 3.01E-05 |
| 10:8:2 Decane : HFE7500 : FC43 | 0.730 | 1.614:  1.860 | 0.8374 | 2.20E-05 | 1.5167 | 6.21E-05 |
| 10:7:3 Decane : HFE7500 : FC43 | 0.730 | 1.614:  1.860 | 0.8295 | 7.39E-05 | 1.5423 | 4.37E-05 |
| 10:6:4 Decane : HFE7500 : FC43 | 0.730 | 1.614:  1.860 | 0.8064 | 4.00E-05 | 1.6114 | 5.35E-06 |

## Supplementary Table S3: UCST For oil combinations used in the paper

| Oil combination | Upper critical solution temperature (°C) |
| --- | --- |
| Decane : Methoxyperfluorobutane | 28.6 |
| Diethylbenzene : HFE7500 | 45.0 |
| Decane : HFE7500 | 38.7 |
| 10:9:1 Decane : HFE7500 : FC43 | 48.4 |
| 10:8:2 Decane : HFE7500 : FC43 | 56.1 |
| 10:7:3 Decane : HFE7500 : FC43 | 64.2 |
| 10:6:4 Decane : HFE7500 : FC43 | 79.0 |

## Supplementary Table S4: Interfacial tensions of hydrocarbon-fluorocarbon oil mixtures

| Oil combination | Interfacial tension (mN/m) | Standard deviation |
| --- | --- | --- |
| Decane : Methoxyperfluorobutane | 0.09 | 0.00224 |
| Diethylbenzene : HFE7500 | 0.57 | 0.00976 |
| Decane : HFE7500 | 0.41 | 0.00842 |
| 10:9:1 Decane : HFE7500 : FC43 | 0.70 | 0.0267 |
| 10:8:2 Decane : HFE7500 : FC43 | 0.91 | 0.0267 |
| 10:7:3 Decane : HFE7500 : FC43 | 1.23 | 0.04761 |
| 10:6:4 Decane : HFE7500 : FC43 | 1.80 | 0.11059 |

# Supplementary Figures


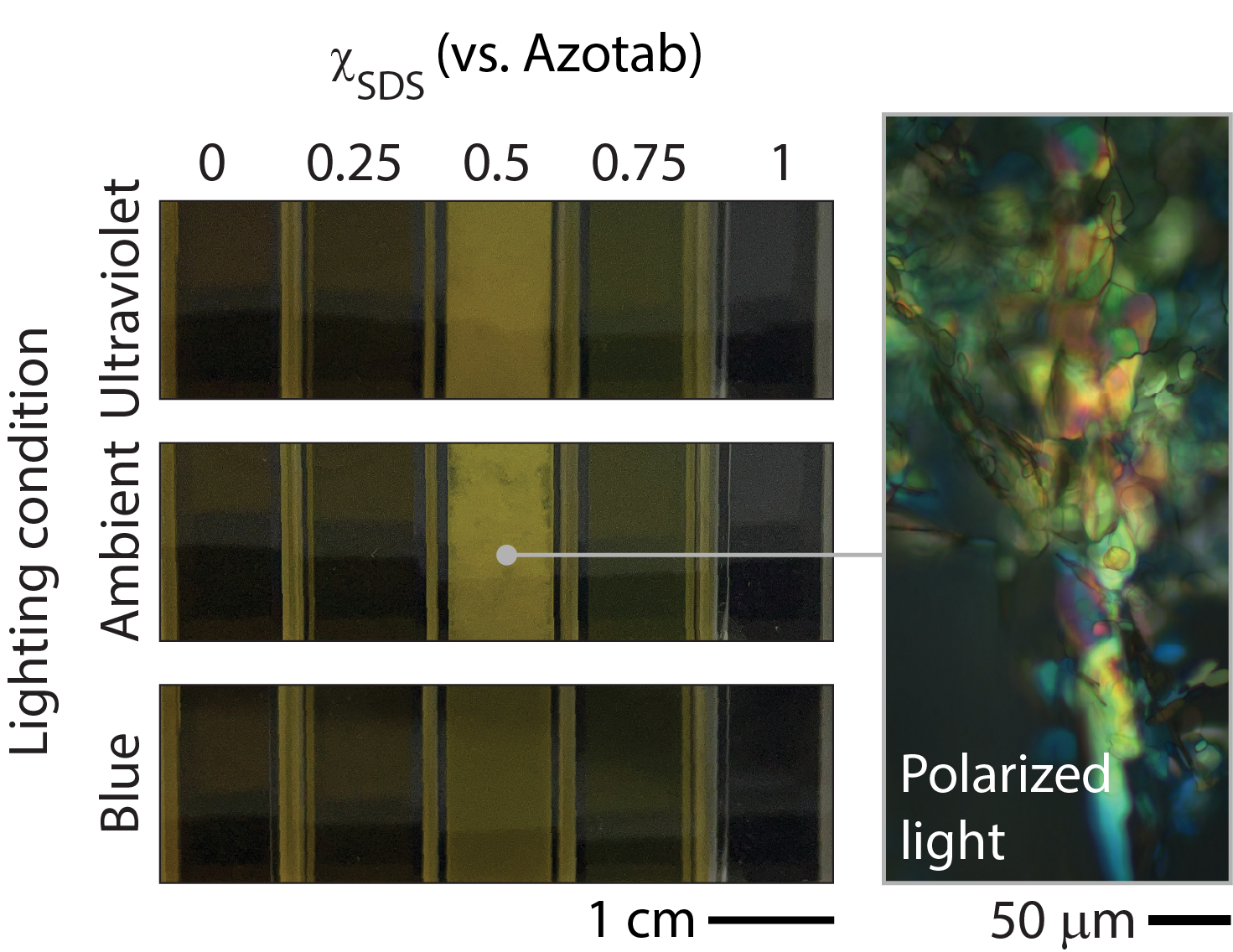


**Supplementary** Figure S1. Photographs of cuvettes containing various molar fractions of SDS and AzoTAB (total concentration 5 mM) after 1 hour, and the same vials after 1 hour of either ultraviolet or blue light application, with inset polarized light micrograph of equimolar AzoTAB:SDS in ambient conditions.


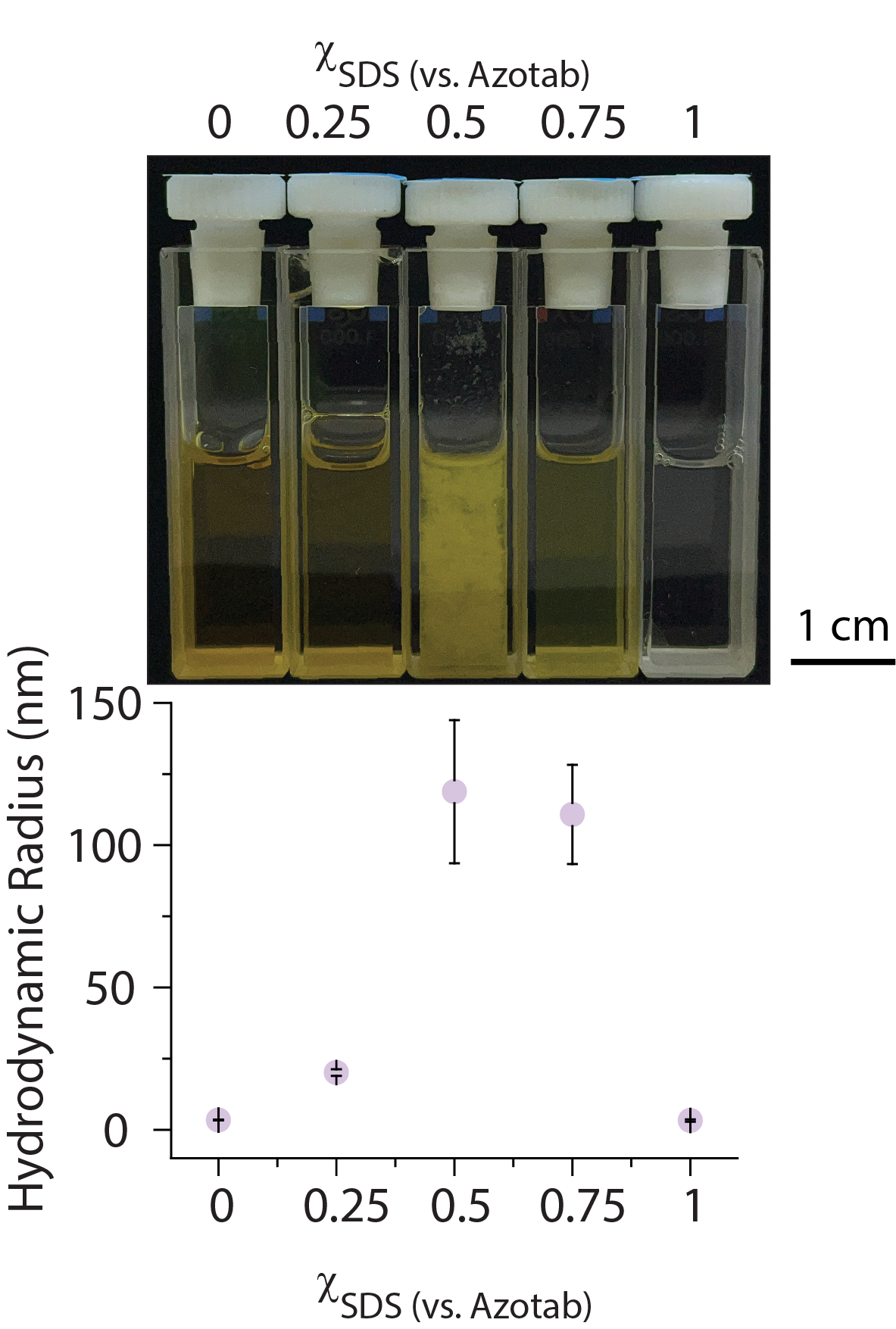


**Supplementary** Figure S2. The precipitate forms at room temperature and ambient lighting conditions for certain fractions of SDS and AzoTAB, visible in fractions 0.5:0.5 SDS:AzoTAB, and 0.75:0.25 SDS:AzoTAB.


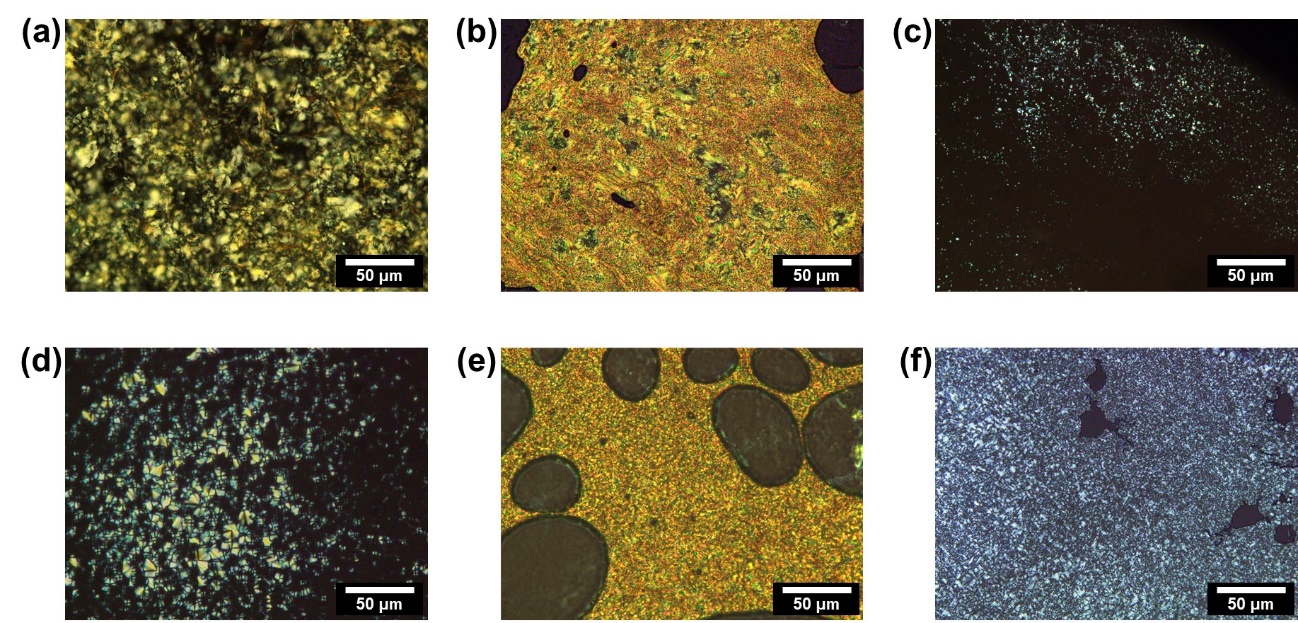


**Supplementary** Figure S3. Polarized optical microscopy (POM) images of AzoTAB-SDS reveal distinct temperature-dependent phase behaviors. Initially, at room temperature, the precipitated material takes the form of a crystalline solid (**a**). Upon heating to 110 ⁰C, the crystalline material transforms into a birefringent fluid phase with loosely defined birefringent textures (b**),** ultimately becoming isotropic at 190 ⁰C. During the cooling process from the isotropic liquid, at 180 ⁰C, the liquid crystal phase appears as nematic droplets (**c**) that merge (**d**), leading to the formation of the nematic liquid crystal phase (**e**). Finally, at 80 ⁰C, the nematic liquid crystal phase undergoes crystallization (**f**).


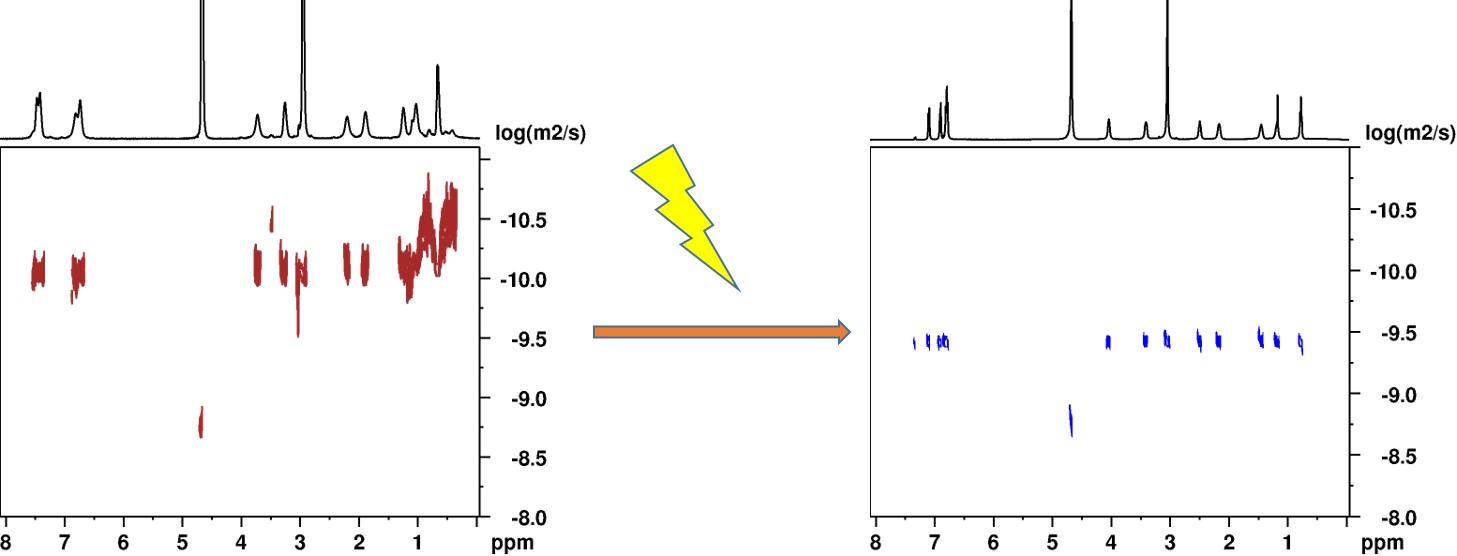


**Supplementary F**igure S4. DOSY experiments of *trans* (in red) and *cis* (in blue) AzoTAB-SDS complex in a molar ratio 10:1.

**Supplementary F**igure S5. ^1^H-^1^H NOESY experiment of *trans* AzoTAB-SDS complex in a molar ratio 10:1.(t_mix_= 0.1s)


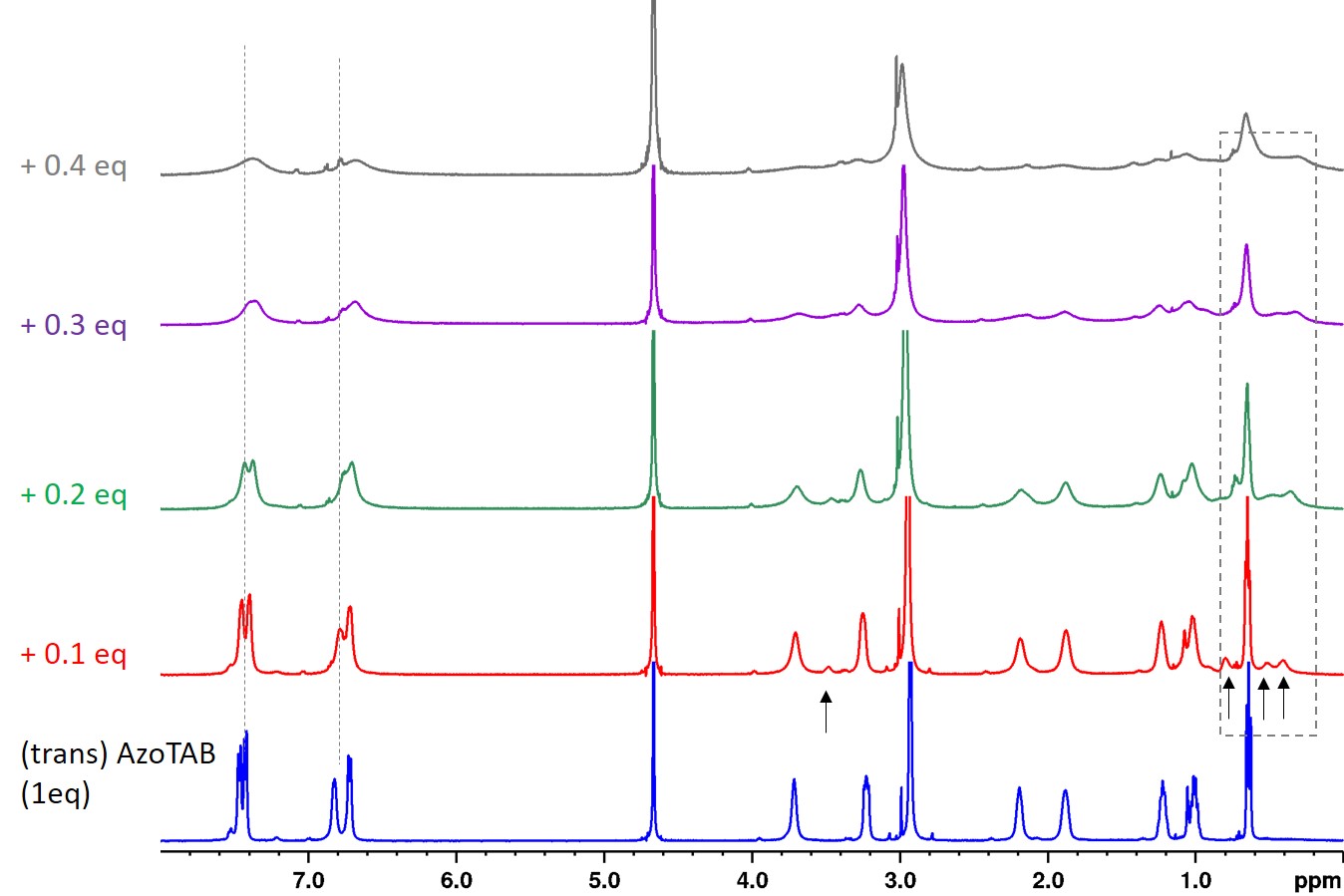


**Supplementary** Figure S6. ^1^H NMR titration of *trans* AzoTAB with SDS. The arrows indicate the signals of SDS, which become shielded with increasing SDS concentration due to the integration of SDS molecules within AzoTAB micelles (vide infra). It is noteworthy that increasing amounts of SDS lead to additional broadening of the signals, a consequence of the increased size of the generated micelle, surpassing the limits of NMR studies. Additionally, these micellar solutions precipitate over time.


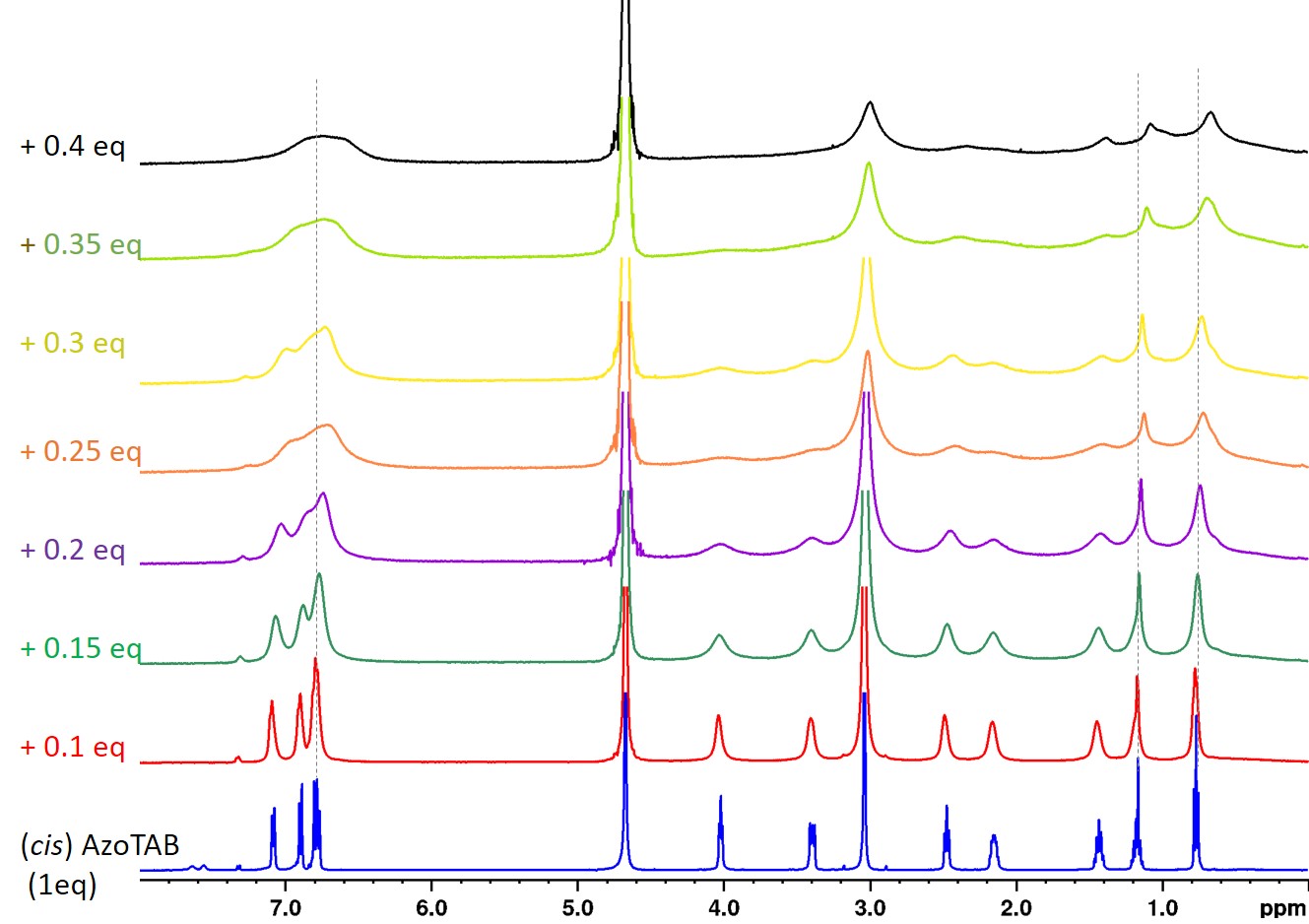


**Supplementary** Figure S7. ^1^H NMR titration of *cis* AzoTAB with SDS. SDS signals are not observable due to overlap with AzoTAB signals. Despite a greater diffusion coefficient compared to trans AzoTAB, indicating the formation of smaller monomeric species (vide infra), a weak association is observed due to π–π interactions. These interactions cause both aromatic protons and those of the alkyl chain of AzoTAB to be shielded with increasing SDS concentration, leading to the precipitation of the complex as titration progresses.

**Supplementary** Figure S8. AzoTAB trans-cis isomerization upon UV-irradiation. Time-dependent ^1^H NMR spectra of SDS at 0.04 wt.% and AzoTAB at 0.15 wt.% in D_2_O after different UV irradiation times. Initially, AzoTAB forms micelles (0.15 wt.% is well above its CMC) that incorporate SDS molecules, as also observed in our NOESY studies (Figure S5). After UV light irradiation, these micelles break apart, resulting in free (monomeric) surfactants in solution. Higher concentrations resulted in a very turbid solution, which could not be measured by NMR due to extremely broad signals.


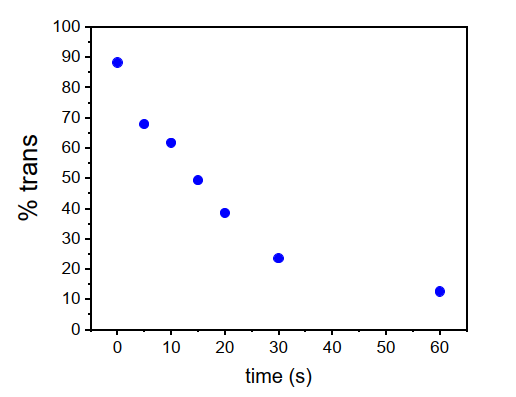


**Supplementary** Figure S9. AzoTAB trans-cis isomerization upon UV-irradiation. As confirmed by time-dependent ^1^H NMR (Figure S8). Almost complete trans-to-cis isomerization occurs in less than 1 minute.


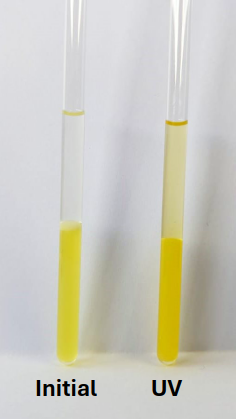


**Supplementary F**igure S10. Visible change in phase partitioning of the SDS-AzoTAB assembly under different lighting conditions. Vials contain 0.3 mL of a D_2_O surfactant solution (SDS at 0.04 wt.% and AzoTAB at 0.15 wt.%), and 0.3 mL of deuterated benzene (C6D6) and the mixture was hand-shaken for 1 minute. The left vial was kept under ambient light conditions, while the right vial was irradiated with UV light for 5 minutes.

**Supplementary F**igure S11. 1H-NMR analysis of a biphasic oil-water mixture containing SDS-AzoTAB assemblies, confirming the shift in surfactant partitioning upon UV light irradiation. In our experiments we added 0.3 mL of deuterated benzene (C_6_D_6_) to 0.3 mL of a D_2_O surfactant solution (SDS at 0.04 wt.% and AzoTAB at 0.15 wt.%), and the mixture was hand-shaken for 1 minute. After 15 minutes of equilibration, ^1^H NMR experiments were conducted separately for both the D_2_O and C_6_D_6_ phases. Under ambient light conditions, no changes were observed in the ^1^H NMR spectrum compared to the initial one. In the C_6_D_6_ phase no surfactants were detected, and the solution remained colorless. After irradiation with UV light for 5 minutes followed by 15 minutes of equilibration, 1H NMR spectra show significant changes. AzoTAB-SDS was now detected in the oil phase. Interestingly, the solution also turned orange (Figure S10). This phase likely contains a mix of cis and some trans free monomeric surfactants, along with corresponding inverted micelles.


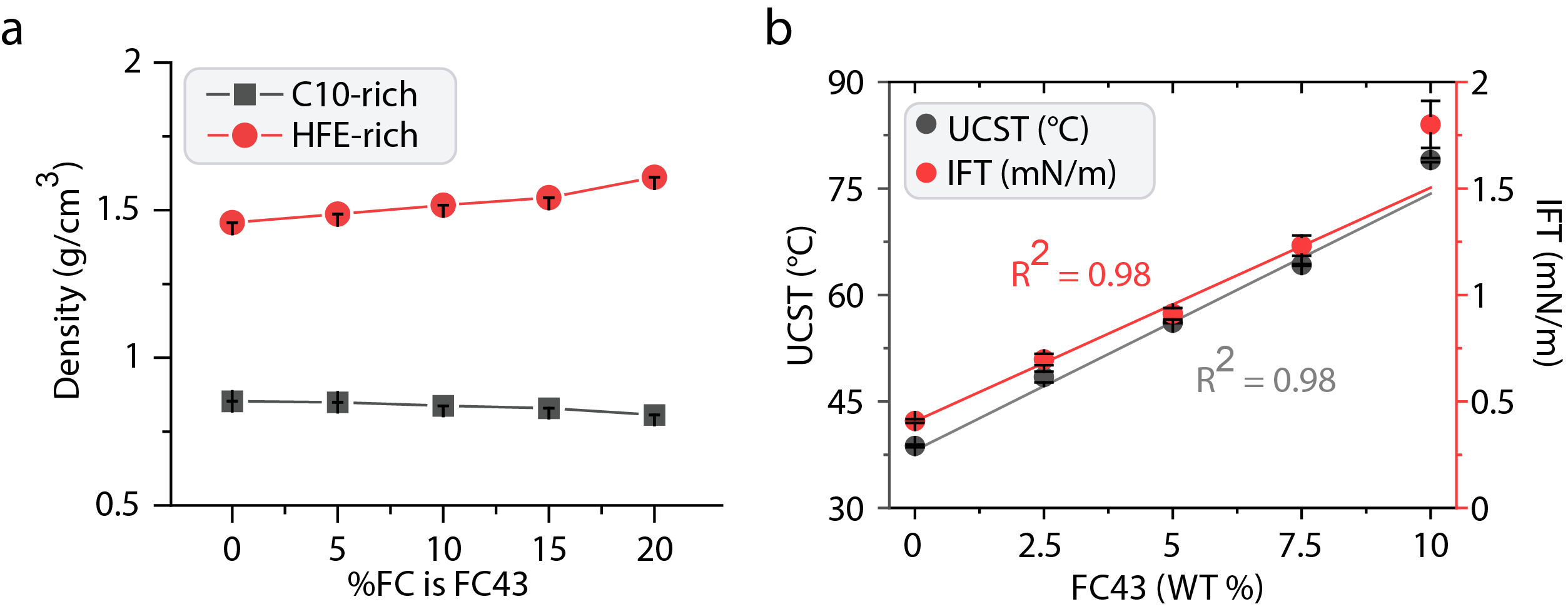


**Supplementary** Figure S12**.** a) Density of both the hydrocarbon and fluorocarbon phase versus the fraction of FC43 within the fluorocarbon phase of the droplet, as the fraction of FC43 increases not only does the density of the fluorocarbon phase lower, but the raising of the upper critical solution temperature of the system decreases miscibility, decreasing the fluorocarbon fraction within the hydrocarbon phase, and therefore decreasing the density. b) Interfacial tension (red) versus the upper critical solution temperature (gray) for mixtures of decane and HFE with increasing volumes of FC43 added as measured with spinning drop tensiometry


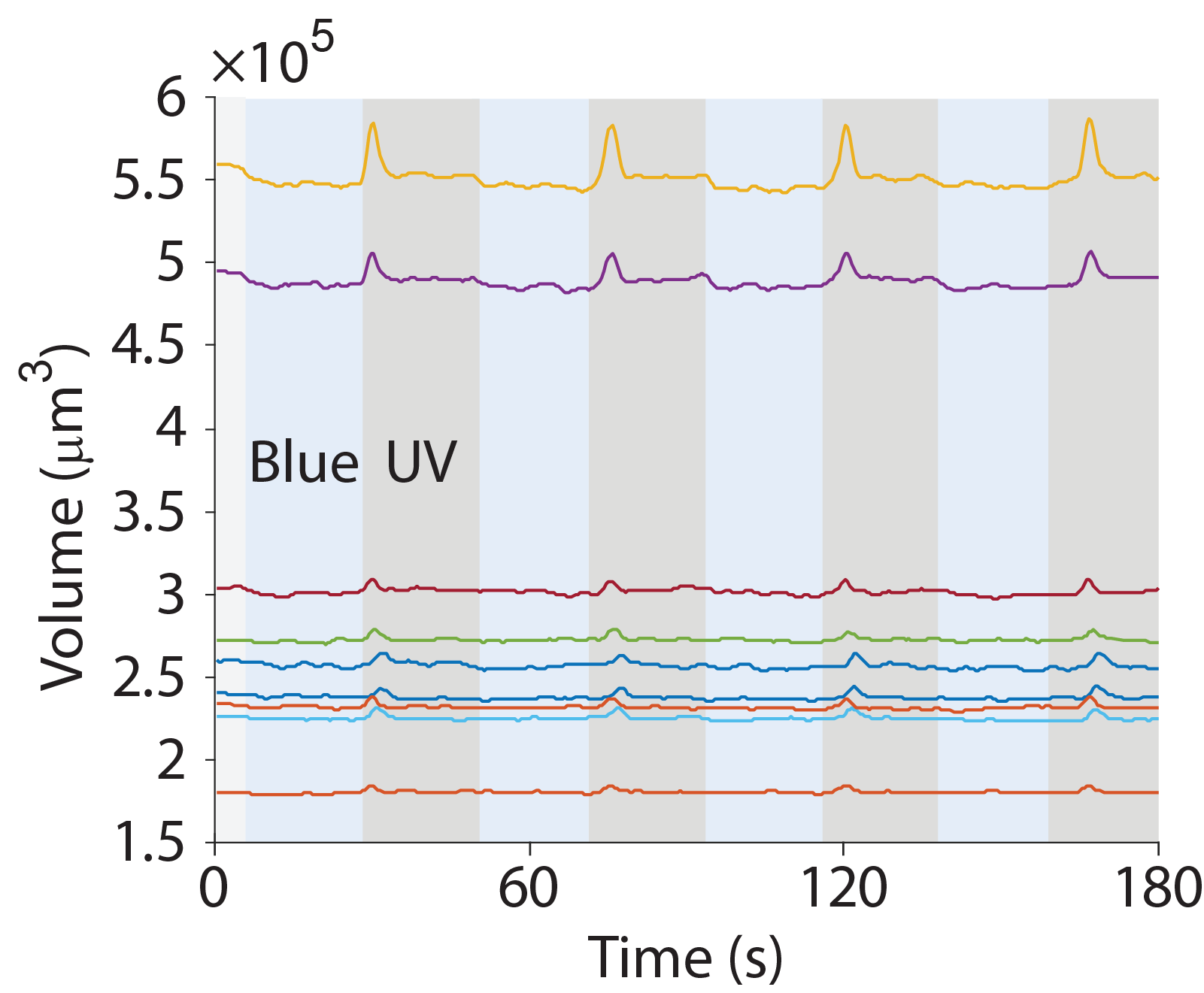


**Supplementary** Figure S13. Volume versus time for emulsion droplets composed of octane and HFE7500 of various sizes, placed in SDS:AzoTAB, and tracked for their change of volume over time, with the application of Blue (shaded blue) light to the sample, or Ultraviolet (UV) light, shaded gray.

**
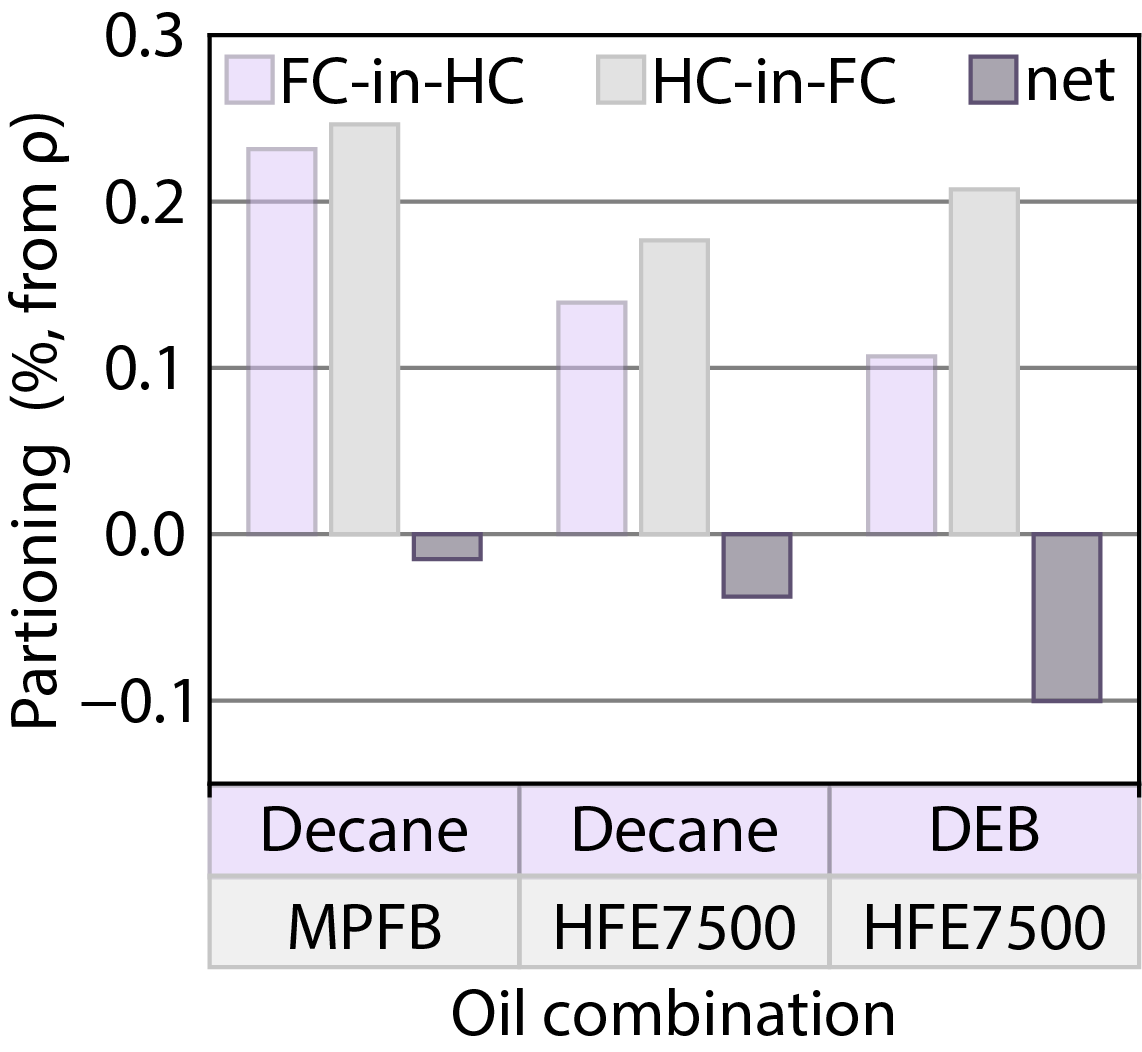
**

**Supplementary** Figure S14**.** Measurements of oil partitioning for 3 droplet oil combinations as measured by densiometry, indicating the magnitude of oil partitioning into hydrocarbon and fluorocarbon-rich oil phases.

**
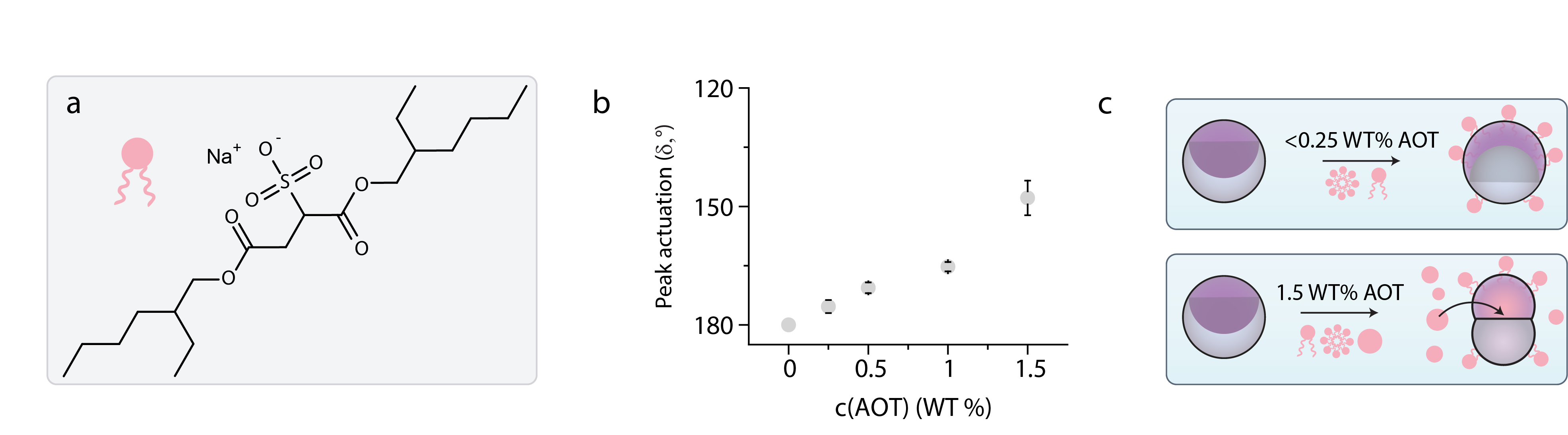
**

**Supplementary** Figure S15. A Janus droplet can be actuated out of sphericity by the mutual lowering of external interfacial tensions, and raising of internal interfacial tensions, we replicate these results with a single component. Dioctyl sodium sulfosuccinate (a) at high concentrations will prefer to spontaneously emulsify rather than solubilize,^44^ as well as the double-hydrocarbon chain, and will stabilize hydrocarbon-water and fluorocarbon-water interfaces.^15^ By placing droplets in increasing concentrations of AOT, droplets uptake surfactant as well as experience lowered hydrocarbon-water and fluorocarbon-water interfacial tensions, generating non-spherical emulsions with tunable peak actuations, measured as the external contact angle (δ) of droplets composed of decane and HFE in varying concentrations of AOT after five minutes (b). This effect is due to the internal interfacial tension raising and external lowering leading to non-spherical emulsion droplets on the minute timescale (c).


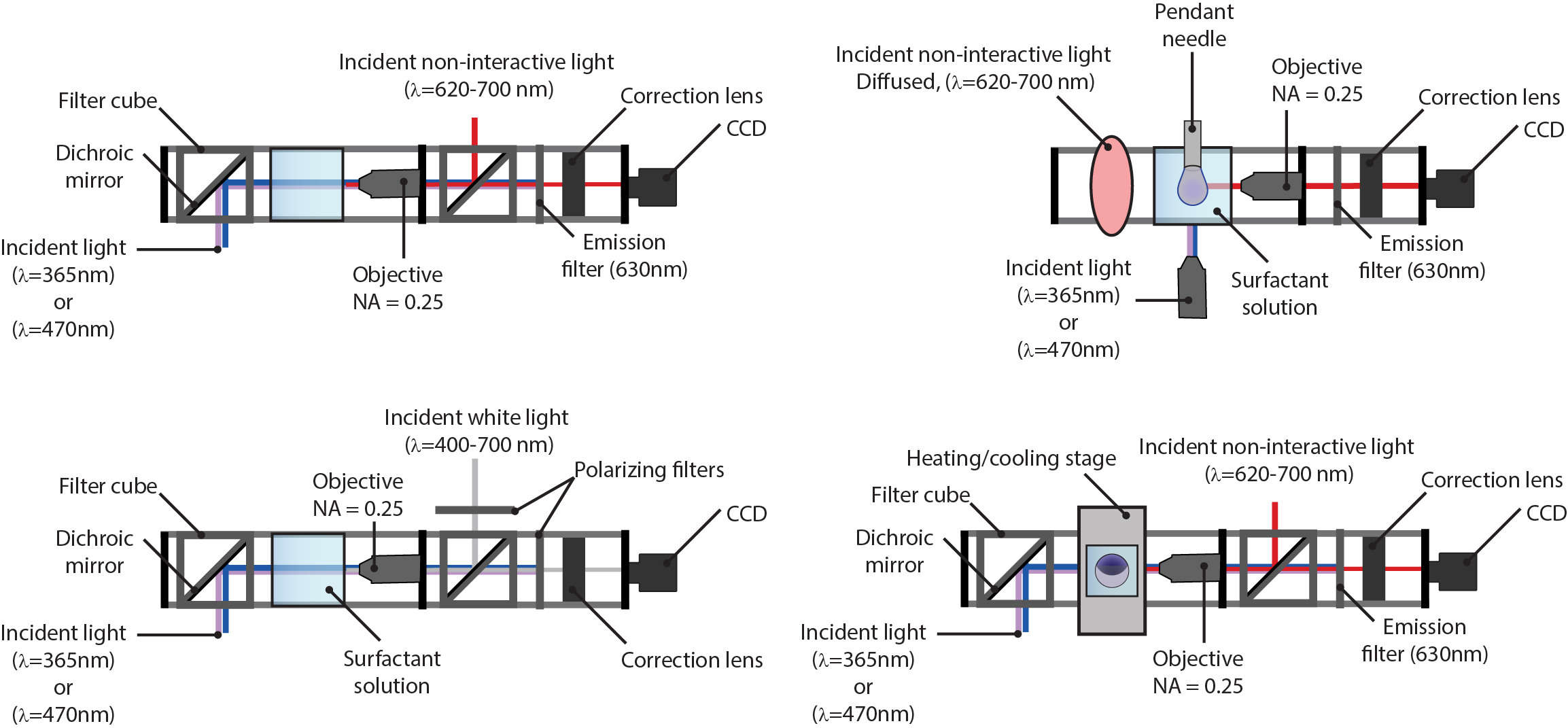


**Supplementary** Figure S16. Description of various optical setups used within the paper. A reconfigurable, rotating microscope was used with particular modification for each experiment. The primary feature of the microscope is the ability to both changing lighting modes, and the orientation between sideview and top view microscopy. For photo-sensitive with switching ultraviolet/blue light, a beam splitter and red filter were used to apply blue/ultraviolet light to the sample and image red light. This concept was used also for pendant drop experiments, with the exception that light was delivered from the side of the sample separate from the microscope.

**
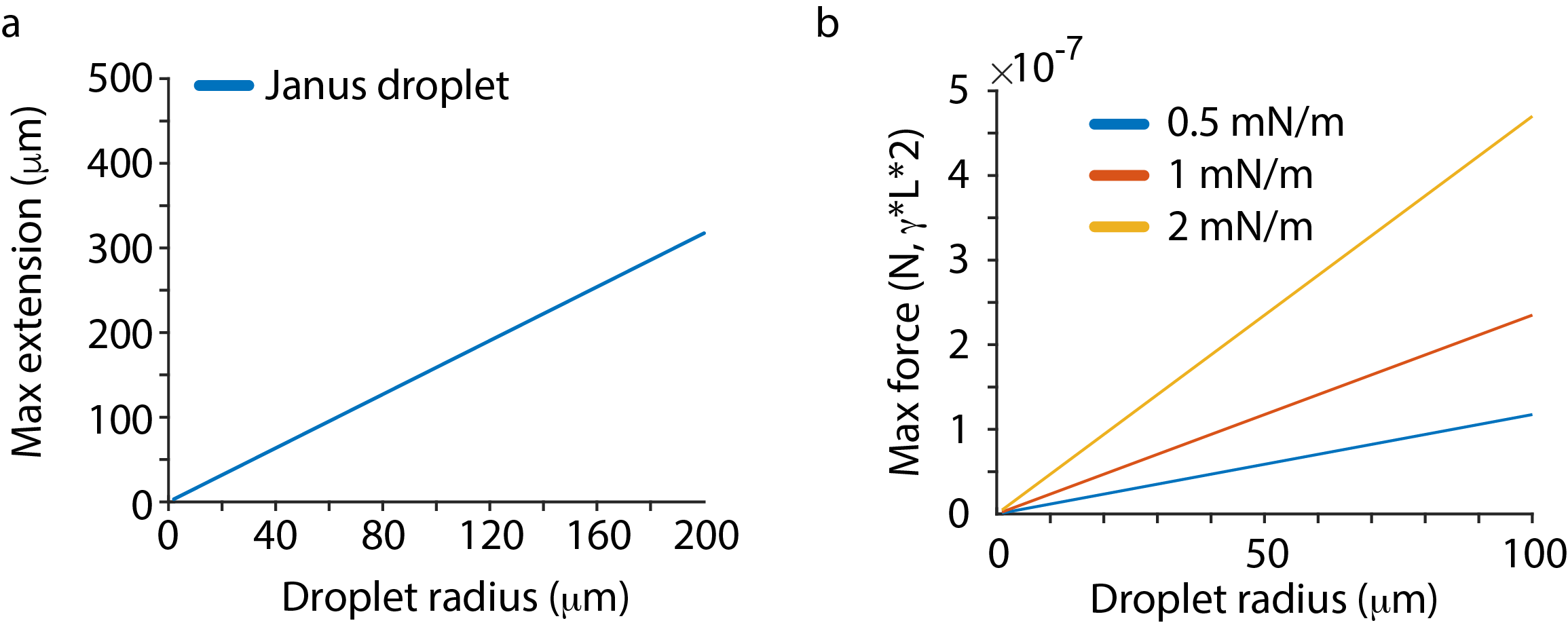
**

**Supplementary** Figure S17. Estimated linear actuating force for a Janus droplet with equal volume hemispheres given a minimum interfacial tension in the system. a) Estimated maximum extension in micrometers for a Janus droplet transforming from a spherical form with radius (x axis) to the estimation of two equal volume spheres just before disconnection. b) Estimation of the maximum linear force generated by the extension of a spherical Janus droplet, which increases per the droplets radius due to the increase in resisted length by the object.

**
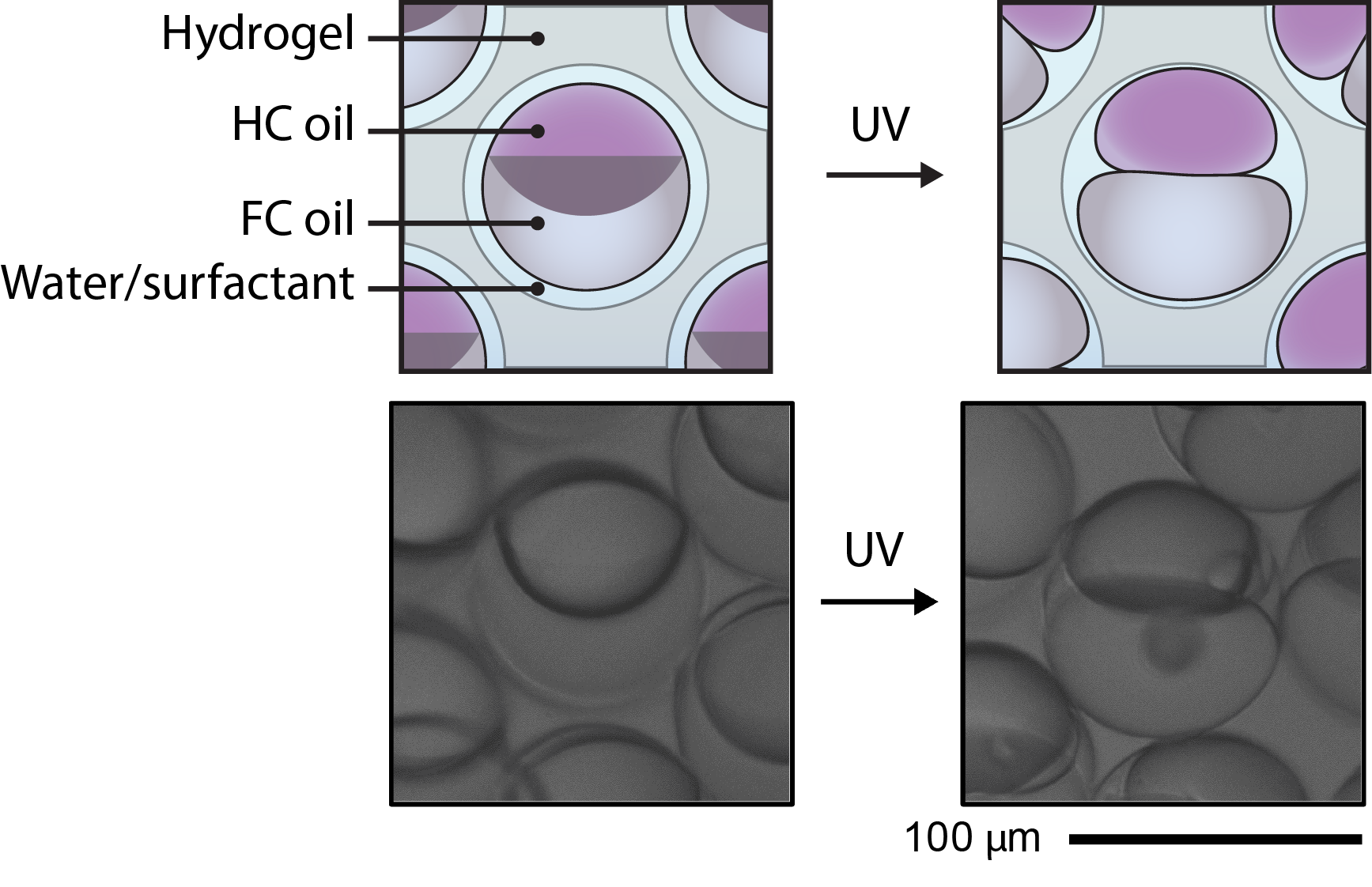
**

**Supplementary** Figure S18. Droplets separate irreversibly in confinement when one surfactant is above the critical micelle concentration. Complex emulsion droplets composed of decane and HFE-7500 are generated with microfluidics, before being stabilized in a solution containing 1 WT% PEG-Diacrylate (M_w_ ~700), and 1 WT% PEG Methyl ether acrylate (M_w_ ~428), with 1 WT % Irgacure 2959, and 1 WT % Zonyl FS-300. The solution is polymerized via ultraviolet light to generate the hydrogel shell, before the hydrogel is swollen by diluting the surfactant in solution to 0.25 WT %, and adding equimolar SDS and AzoTAB at 2.5 mM. On the application of ultraviolet light, the hydrocarbon and fluorocarbon phases separate into separate single-phase emulsion droplets which are deformed when retained in confinement.

# Supporting Videos

Supporting Video 1. Droplets composed of decane and HFE:7500 actuate in a solution of AzoTAB and sodium dodecyl sulfate, under the response of UV light, or blue light as noted. The video is in real time with noted scalebar.

Supporting Video 2. An equimolar solution of sodium dodecyl sulfate and AzoTAB are observed under polarized light microscopy on the application of ultraviolet and blue light, where the surfactant precipitate is observed to crystalize under blue light, and form coascervate under ultraviolet light. The video is in real time with noted scalebar.

Supporting Video 3. Droplets composed of octane and HFE7500 in an equimolar solution of AzoTAB and SDS (2.5 mM) are observed under the application of blue or ultraviolet light, a change in droplet radius is observed under the application of either ultraviolet or blue light, where the droplet is observed to grow with ultraviolet light and quickly shrink under the application of blue light. The video is in real time with noted scalebar.

Supporting Video 4. Decane-rich sessile droplet actuates out of sphericity with the application of ultraviolet light over a period of 10 seconds. The video is in real time with noted scalebar.

Supporting Video 5. A droplet in a well cuvette movies via wall-pushing due to the changing radius of the overall droplet when actuating between spherical and non-spherical shapes, video is in real time with noted scalebar.

Supporting Video 6. Droplets composed of diethylphthalate, dibromomethane, and HFE7500 with equal density hydrocarbon and fluorocarbon phases sit on their side, and by actuating the droplets with ultraviolet or blue light can be moved via crawling on the glass slide. The video is in real time with noted scalebar.

Supporting Video 7. Water-in-oil-in-water double emulsions composed of a water phase which contains tracer particles, and an oil phase composed of HFE7500 are subjected to ultraviolet light in a solution of AzoTAB and SDS, releasing the cargo into the aqueous phase. The video is in real time with noted scalebar.

Supporting Video 8. A droplet composed of diethylphthalate, dibromomethane, and HFE7500 is directed to achieve several behaviors in a solution of AzoTAB and SDS as noted in the video, including destabilization, re-stabilization, separation, and further re-separation, along with directed crawling. The video is in real time with noted scalebar.

Supporting Video 9. Droplets composed of diethylbenzene : HFE7500, decane : HFE7500 and decane : methoxyperfluorobutane are actuated in a solution of AzoTAB and SDS, in uniform lighting conditions at uniform concentrations.

# References

1. Zarzar LD, Sresht V, Sletten EM, Kalow JA, Blankschtein D, Swager TM. Dynamically reconfigurable complex emulsions via tunable interfacial tensions. *Nature* **518**, 520-524 (2015).

2. Wang J, Hahn S, Amstad E, Vogel N. Tailored Double Emulsions Made Simple. *Adv Mater*, e2107338 (2021).

3. Chevallier E, Mamane A, Stone HA, Tribet C, Lequeux F, Monteux C. Pumping-out photo-surfactants from an air–water interface using light. *Soft Matter* **7**, 7866-7874 (2011).

4. Djalali S, Frank BD, Zeininger L. Responsive drop method: quantitative in situ determination of surfactant effectiveness using reconfigurable Janus emulsions. *Soft Matter* **16**, 10419-10424 (2020).

5. Zeininger L*, et al.* Rapid Detection of Salmonella enterica via Directional Emission from Carbohydrate-Functionalized Dynamic Double Emulsions. *ACS Cent Sci* **5**, 789-795 (2019).

6. Guzowski J, Korczyk PM, Jakiela S, Garstecki P. The structure and stability of multiple micro-droplets. *Soft Matter* **8**, 7269-7278 (2012).

7. Crocker JC, Grier DG. Methods of digital video microscopy for colloidal studies. *J Colloid Interface Sci* **179**, 298-310 (1996).

8. Huang E*, et al.* OpenDrop: Open-source software for pendant drop tensiometry & contact angle measurements. *Journal of Open Source Software* **6**, 2604 (2021).

9. Atefi E, Mann JA, Jr., Tavana H. Ultralow Interfacial Tensions of Aqueous Two-Phase Systems Measured Using Drop Shape. *Langmuir* **30**, 9691-9699 (2014).

10. Saad SMI, Neumann AW. Axisymmetric Drop Shape Analysis (ADSA): An Outline. *Adv Colloid Interface Sci* **238**, 62-87 (2016).

11. Ijavi M*, et al.* Surface tensiometry of phase separated protein and polymer droplets by the sessile drop method. *Soft Matter* **17**, 1655-1662 (2021).
